# SIN-3 acts in distinct complexes to regulate the germline transcriptional program in *Caenorhabditis elegans*

**DOI:** 10.1242/dev.201755

**Published:** 2023-10-17

**Authors:** Valerie J. Robert, Matthieu Caron, Loic Gely, Annie Adrait, Victoria Pakulska, Yohann Couté, Manon Chevalier, Christian G. Riedel, Cecile Bedet, Francesca Palladino

**Affiliations:** ^1^ Laboratory of Biology and Modeling of the Cell, UMR5239 CNRS/Ecole Normale Supérieure de Lyon, INSERM U1210, UMS 3444 Biosciences Lyon Gerland, Université de Lyon, 69007 Lyon, France; ^2^ Grenoble Alpes, CEA, Inserm, UA13 BGE, CNRS, CEA, FR2048, 38000 Grenoble, France; ^3^ Department of Biosciences and Nutrition, Karolinska Institutet, Blickagången 16, 14157 Huddinge, Sweden

**Keywords:** HDAC, SIN3, X chromosome, *C. elegans*, Germline, Proteomics

## Abstract

The transcriptional co-regulator SIN3 influences gene expression through multiple interactions that include histone deacetylases. Haploinsufficiency and mutations in *SIN3* are the underlying cause of Witteveen–Kolk syndrome and related intellectual disability and autism syndromes, emphasizing its key role in development. However, little is known about the diversity of its interactions and functions in developmental processes. Here, we show that loss of SIN-3, the single SIN3 homolog in *Caenorhabditis elegans*, results in maternal-effect sterility associated with de-regulation of the germline transcriptome, including de-silencing of X-linked genes. We identify at least two distinct SIN3 complexes containing specific histone deacetylases and show that they differentially contribute to fertility. Single-cell, single-molecule fluorescence *in situ* hybridization reveals that in *sin-3* mutants the X chromosome becomes re-expressed prematurely and in a stochastic manner in individual germ cells, suggesting a role for SIN-3 in its silencing. Furthermore, we identify histone residues whose acetylation increases in the absence of SIN-3. Together, this work provides a powerful framework for the *in vivo* study of SIN3 and associated proteins.

## Teaser

**Highlighted Article:** SIN3 complexes are required for fertility in *C. elegans* and contribute to repression of X-linked genes in the germline.

## INTRODUCTION

The highly conserved transcriptional co-regulator SIN3 acts as a scaffold to assemble distinct complexes containing histone deacetylases (HDACs), chromatin adaptors and transcription factors that modify chromatin to influence gene expression (Laherty et al., 1997; Banks et al., 2018, 2020). SIN3/HDAC complexes regulate essential cellular processes, including differentiation, cell cycle regulation and metabolism through both gene activation and repression (Saha et al., 2016; van Oevelen et al., 2010). Importantly, heterozygous loss-of-function variants and point mutations in the two mammalian homologs *SIN3A* and *SIN3B* were recently identified as the underlying cause of Witteveen–Kolk syndrome and related intellectual disability disorders (Balasubramanian et al., 2021; Latypova et al., 2021; Witteveen et al., 2016), emphasizing the functional importance of these proteins.

In different species, SIN3 proteins reside in multiple complexes. In yeast, these are named Rpd3 large (Rpd3L: Sin3, Rpd3/HDA1, Ume1, Sap30, Sds3/SUDS3, and other proteins) and Rpd3 small (Rpd3S: Sin3, Rpd3/HDA1, Ume1, Rco1 and Eaf3) (Carrozza et al., 2005a,b; Kadosh and Struhl, 1997; Kasten et al., 1997). Mammalian SIN3A and SIN3B share overall domain structure and association with HDAC1 and HDAC2. Biochemical data from numerous studies suggests that they function in distinct complexes, and that paralog identity influences complex composition, with SIN3B co-purifying in a complex equivalent to yeast Sin3S/Rpd3S as well as larger complexes (Jelinic et al., 2011; Vermeulen et al., 2010; Adams et al., 2020; Banks et al., 2018, 2020) and SIN3A residing in multiple large complexes related to yeast SIN3L/Rpd3L (Banks et al., 2018, 2020; Streubel et al., 2017; Saunders et al., 2017; Fleischer et al., 2003; Alland et al., 2002; Zhang et al., 1997; Adams et al., 2020). Both homologs interact directly with transcription factors through one of three paired-amphipathic helix (PAH) domains, and are recruited to chromatin through accessory proteins including ARID4A/B, PHF12/PF1 and MRG15. Mouse knockout studies have shown that SIN3A and SIN3B are non-redundant (Dannenberg et al., 2005; David et al., 2008), consistent with at least partially distinct functions. However, the presence of two homologs, the temporal and cell type-specific nature of SIN3 complex activities, and the transient association of SIN3 with numerous transcription factors and accessory proteins has hampered the study of individual complexes in a developmental context.

*Caenorhabditis elegans* contains a single SIN3 homolog, SIN-3, facilitating its study (Choy et al., 2007; Beurton et al., 2019). Genetic analysis of *sin-3* mutant animals carrying the molecularly uncharacterized *tm1276* allele revealed roles in male development (Choy et al., 2007), motility, longevity and fertility (Pandey et al., 2018; Sharma et al., 2018), but the specific function of SIN-3 in these different processes remains mostly unknown, as does the identity of its interaction partners. Here, we combined genetic analysis with affinity purification coupled to mass spectrometry (MS)-based proteomics, transcriptomics and single-cell analysis to dissect more clearly the function of SIN-3. Using a *sin-3* null mutant constructed using CRISPR-Cas9, we uncovered an essential requirement for SIN-3 in germ stem cell proliferation and fertility. Immunoprecipitation followed by MS-based proteomic (IP-MS) analysis confirmed the identity of the previously described SIN3 small (SIN3S) complex containing MRG-1/MRG15 (MORF4L1), HDA-1/HDAC and ATHP-1/PHF12 (Beurton et al., 2019). In addition, we identified counterparts of known mammalian SIN3L complex subunits, including SUDS-3/SUDS3 and ARID-1/ARID4, thereby defining at least two distinct SIN3 complexes: SIN3S and SIN3L. We also provide evidence that specific HDACs reside in different SIN3 complexes, and that these differentially contribute to germline health. Genome-wide transcriptomics analysis combined with single-molecule inexpensive fluorescence *in situ* hybridization (smiFISH; Tsanov et al., 2016) reveals that loss of SIN-3 results in de-silencing of X-linked genes in a stochastic manner, and histone post-translational modification analysis by MS-based proteomics identifies histone H3K18AcK23Ac as a target of SIN-3-dependent deacetylation. Together, our results reveal an essential role for SIN-3 in preserving the germline transcriptional program and fertility, and provide insight on how, within a single tissue, distinct SIN3 complexes and interaction partners contribute to specific regulatory functions.

## RESULTS

### Loss of *sin-3* results in maternal effect sterility

**Fig. 1.**
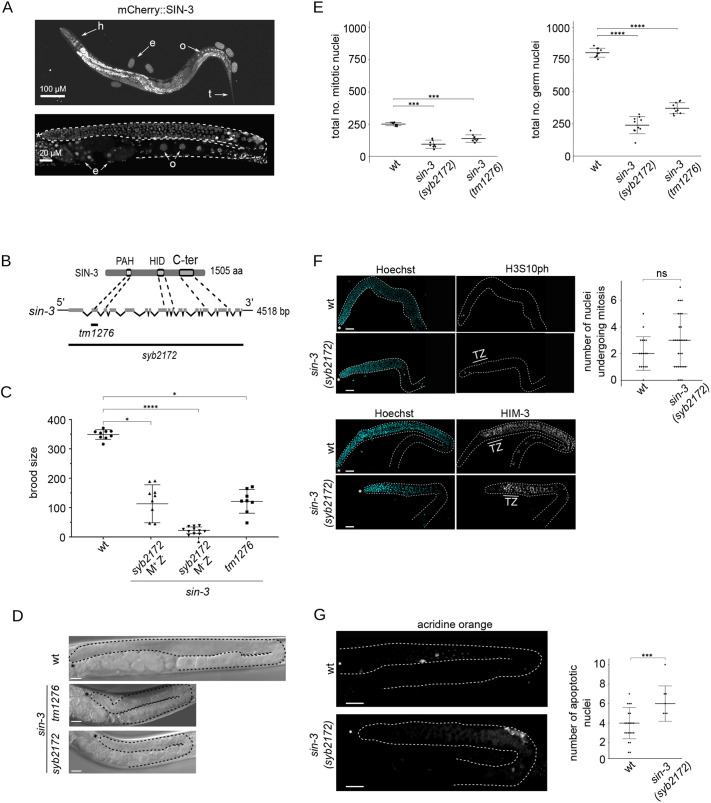
**Sterility and reduced progenitor cell number in *sin-3* deletion mutants.** (A) Representative image of a whole animal expressing endogenously tagged mCherry::SIN-3. Top: Expression of SIN-3 throughout the body including nuclei in head (h) and tail (t), embryos (e) and oocytes (o). Final image was obtained by stitching each image to its neighbors with the best alignment. Bottom: SIN-3 expression throughout the adult germline (one arm of the gonad is shown, outlined with dashed line). Asterisk marks the distal end of the germline. Images were taken from different animals. (B) Schematic of the SIN-3 protein with conserved domains and the corresponding exon sequence on the *sin-3* gene. The extent of the *tm1276* and *syb2172* deletions are indicated below the gene sequence. (C) Brood size count of wild type (wt) and *sin-3* mutants. M+Z−, homozygous mutant with maternal contribution; M−Z−, homozygous mutant without maternal contribution. Multiple comparison was performed using Dunn's multiple comparisons test following a significant Kruskal–Wallis test; *P<0.05; ****P<0.0001. (D) Representative differential interference contrast microscopy images of germlines from wild-type and *sin-3* mutant animals. Germlines are outlined with dashed lines; asterisks mark the distal end of the germline. (E) Total number of germline nuclei, and number of mitotic nuclei per gonad arm in wild-type and *sin-3* mutant worms. Ten gonad arms were counted per genotype. Multiple comparison was performed using a generalized linear model with a quasi-Poisson law. ****P<0.0001. (F) Representative confocal microscopy images of phospho-H3 (upper panels) and HIM-3 (lower panels) immunolabeling on dissected germlines (outlined with dashed lines) from wild-type and *sin-3(syb2172)* animals. For HIM-3 immunolabeling, the transition zone (TZ) defined by Hoechst staining is displayed. Statistical comparison was performed using a Mann–Whitney–Wilcoxon test. *n*=17 for wild type and *n*=30 for *sin-3(syb2172).* (G) Acridine orange (AO) staining of apoptotic cells in a single gonad arm from whole animals. Dot plot displays the number of apoptotic bodies stained with AO per germline. Statistical comparison was performed using one-tailed, unpaired Student's *t*-test. *n*=22 for wild type and *n*=7 for *sin-3(syb2172)*. ***P<0.001. In panels D-G, all *sin-3* mutant animals analyzed are M−Z−. Scale bars: 100 µm (A, top); 20 µm (A, bottom); 10 µm (D,F); 15 µm (G). Error bars in C,F-G represent s.d. ns, not significant.

An mCherry::SIN-3 translational fusion protein constructed by CRISPR-Cas9 is ubiquitously expressed in germline and somatic nuclei, and in embryos starting at the 4-cell stage (Fig. 1A, Fig. S1A). Previous studies using the *sin-3(tm1276)* allele revealed defects in the male tail, decreased lifespan and reduced fertility (Beurton et al., 2019; Choy et al., 2007; Sharma et al., 2018). *tm1276* is a small internal deletion that only removes exon 2 of *sin-3*. We therefore used CRISPR-Cas9 genome editing to construct a full knockout allele, *syb2172*, in which the ATG codon and the entire *sin-3* coding region are removed (Fig. 1B). The *syb2172* allele is maintained over a *sin-3*(+) balancer chromosome (see Materials and Methods). We observed that F1 homozygous *syb2172* progeny derived from heterozygous mothers, which inherit maternal *sin-3*(+) product but do not synthesize zygotic product (abbreviated M+Z−), produce significantly fewer F2 offspring than do wild type (mean 120 versus 350; Fig. 1C). Second-generation F2 animals without maternal contribution (M−Z−) developed into fully sterile adults, with a few animals producing ten or fewer progeny. *sin-3(tm1276)* animals can instead be maintained as homozygotes, although they produce fewer progeny (Beurton et al., 2019; Pandey et al., 2018) (Fig. 1C). Together, these results show that complete loss of *sin-3* causes sterility in absence of maternal contribution and indicate that the original *tm1276* mutation is most likely a partial loss-of-function allele.

### Reduced proliferation of progenitor cells in *sin-3* mutant germlines

In the *C. elegans* germline, meiotic nuclei are arranged in a spatiotemporal order, with the distal end of the gonad containing mitotically proliferating nuclei. Subsequent stages, clearly recognizable by DAPI staining, consist of a ‘transition zone’, where homolog pairing occurs and chromosomes become polarized in a crescent shape, followed by the pachytene stage, during which synapsed chromosomes appear as discrete, parallel tracks. More proximally, nuclei exit pachytene, enter diplotene, and cellularized oocytes containing condensed homologs are formed (Crittenden et al., 2006). Adult germlines of *sin-3* mutant animals were significantly smaller than wild type, with a more severe phenotype observed in *sin-3(syb2172)* compared with *sin-3(tm1276)* (Fig. 1D). Although the overall organization of *tm1276* germlines was similar to wild type, with recognizable germ cells in the distal region and oocytes proximally, numerous abnormalities were observed in F2 M−Z− *sin-3(syb2172)* germlines, including the appearance of large cells resembling oocytes in the bend region, and a highly disorganized proximal region (Fig. 1D). In approximately 20% of these animals, we also observed a ‘Gogo’ phenotype, in which germ cells transition from oocytes to pachytene germ cells and back to oocytes (germ line-oocyte-germ line-oocyte) (Eberhard et al., 2013; Sendoel et al., 2019) (Fig. S1B).

Counting of DAPI-stained progenitor cells in the distal germline, recognized by their morphology (Crittenden et al., 2006), revealed that in both *tm1276* and s*yb2172* mutants germ cell proliferation was severely reduced: the total number of mitotic nuclei decreased from approximately 250 per gonad arm in wild type to 138 in *tm1276* and fewer than 100 in *syb2172* mutants. A similar decrease was observed when counting the total number of germline nuclei (Fig. 1E). Immunolabeling of *sin-3(syb2172)* mutant germlines with antibodies directed against histone H3 phosphorylated on Ser10 (H3S10ph) to mark dividing cells, and HIM-3 to mark meiotic cells (Zetka et al., 1999), revealed no significant difference compared with wild type either in the number of mitotic figures in the distal region, or in HIM-3 immunolabeling beginning at the transition zone (Fig. 1F). Therefore *sin-3(syb2172)* germlines retain an overall distal-proximal organization similar to wild type. Because phospho-H3 positive cells are in metaphase, a decrease in the number of proliferating germ cells in *sin-3* mutants without a corresponding reduction in the number of mitotic figures may stem from an extended block or pause in metaphase (Golden et al., 2000). In *sin-3(syb2172)* germlines, we further observed a small increase in apoptosis (Fig. 1G). Altogether, these results suggest that a decrease in germ cell proliferation, in combination with a small increase in apoptosis, contribute to the sterility of *sin-3* mutants. More subtle defects in germline organization may also contribute to this loss of fertility, as revealed by the ‘Gogo’ phenotype observed in some animals.

### SIN-3 resides in distinct complexes

SIN-3 does not have DNA-binding or enzymatic activities on its own, and its co-regulatory functions are dependent on interactions with its protein partners (Adams et al., 2018; Kadamb et al., 2013). To identify proteins that interact with SIN-3, we performed IP-MS on mCherry::SIN-3-expressing embryos (Fig. S2A). SIN-3 co-precipitated counterparts of yeast and mammalian Rpd3S/SIN3S subunits ATHP-1/PHF12, MRG-1/MRG15 and HDA-1 (Rundlett et al., 1996; Carrozza et al., 2005b; Jelinic et al., 2011), and COMPASS-targeting subunit CFP-1 (Table 1, Table S1), as expected from previous work using CFP-1 as bait (Beurton et al., 2019). We also identified counterparts of conserved mammalian SIN3L complex subunits SUDS-3/SDS3, and the ARID4 homolog ARID-1 (Banks et al., 2018, 2020; Adams et al., 2020). Low abundance peptides corresponding to RBA-1 and LIN-53, the two homologs of the SIN3S and -L complex component RBBP4/7 (Kelly and Cowley, 2013), were also detected. Peptides corresponding to Y67D2.7, distantly related to the SIN3L subunit SAP30, were not present in our IP-MS analysis (Table S1). Other top hits included HDA-3, a second class I HDAC (Shi and Mello, 1998), the histone chaperone NAP-1, identified as a SIN3 interactor in *Drosophila* (Moshkin et al., 2009), and the uncharacterized protein C01G6.5, encoding an ortholog of the mammalian PHD finger/forkhead transcription factor TCF19, a known interactor of the NuRD HDAC complex (Mondal et al., 2020; Sen et al., 2017). Additional IP-MS experiments using as bait the SIN3L complex component ARID-1/ARID4 (Adams et al., 2020; Banks et al., 2018) tagged with GFP (ARID-1::GFP) (Fig. S2A,B) confirmed the presence of SIN-3 in a larger complex distinct from SIN3S and containing SUDS-3, HDA-3, HDA-1, C01G6.5 and NAP-1, in addition to ARID-1 (Table 1, Fig. S2C, Table S1). Peptides corresponding to ATHP-1/PHF12, a unique subunit of SIN3S complexes (Adams et al., 2018, 2020; Banks et al., 2018; Carrozza et al., 2005b), were absent from ARID-1 IP-MS. A number of nematode-specific proteins were also identified in both SIN-3 and ARID-1 IP-MS experiments (Table 1, Fig. S2C).

**Table 1.**
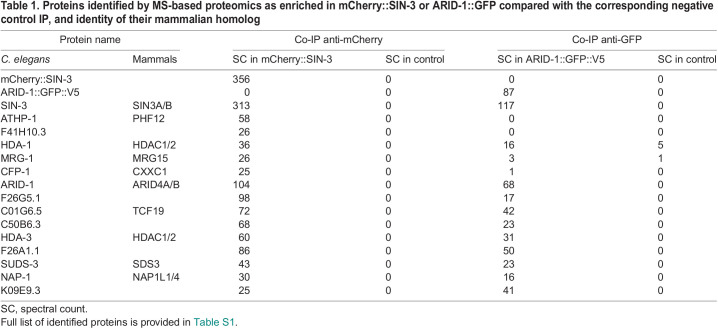
Proteins identified by MS-based proteomics as enriched in mCherry::SIN-3 or ARID-1::GFP compared with the corresponding negative control IP, and identity of their mammalian homolog

**Fig. 2.**
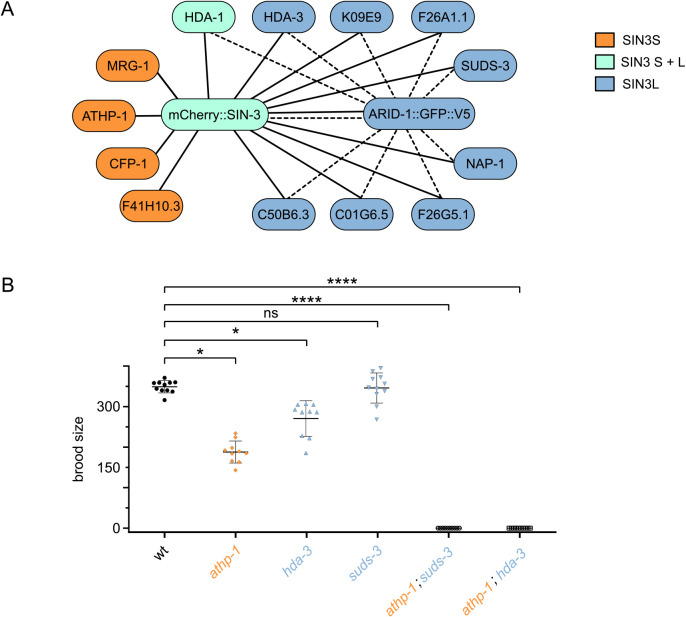
**Identification of two SIN-3 related complexes in *C. elegans* embryos by IP-MS, and impact of inactivation of conserved subunits on fertility.** (A) Schematic of the protein interactions of SIN-3 and ARID-1, and the complexes inferred from them, based on the data in Table 1 and the data of Beurton et al. (2019). Subunits of the SIN3S and SIN3L complexes are orange and blue, respectively; common subunits are turquoise. Solid lines designate SIN-3 interactors, dashed lines ARID-1 interactors. (B) Brood size as a read-out of fertility following inactivation of SIN3L and SIN3S complex subunits. Multiple comparison was performed using Dunn's multiple comparisons test following a significant Kruskal–Wallis test. *P<0.05; ****P<0.0001. ns, not significant. ). Error bars represent s.d.

The above results, together with previous data using either CFP-1 or the SIN3S subunit MRG-1 as bait in IP-MS experiments (Beurton et al., 2019; Baytek et al., 2022), confirm the presence of SIN-3 in a smaller complex related to yeast and mammalian Rpd3/SIN3S (Carrozza et al., 2005b; Jelinic et al., 2011; Adams et al., 2020; Banks et al., 2018, 2020) containing ATHP-1/PHF12, MRG-1/MRG15, CFP-1 and HDA-1 as the only HDAC. In addition, we identify a second complex, which we will hereafter refer to as SIN3L based on its similarity to mammalian SIN3 large complexes (Adams et al., 2020; Banks et al., 2018, 2020), that contains SUDS-3, the class I HDACs HDA-3 and HDA-1, and ARID-1/ARID4. Because neither NAP-1 nor the transcription factor C01G6.5 co-purified with the SIN3S complex in previous experiments (Beurton et al., 2019), we assign them here to a *C. elegans* SIN3L-like complex (Fig. 2A). Furthermore, based on homology with the human protein, we will designate C01G6.5 as TCF-19 in the subsequent text. The absence of COMPASS subunits other than CFP-1 from our list of SIN-3 and ARID-1 interactors confirms the presence of CFP-1 independently of COMPASS in a SIN3S-related complex (Beurton et al., 2019) (Fig. S2C). Our data also suggest that class I HDACs may differentially contribute to the activity of SIN3 complexes: HDA-1 alone in the SIN3S complex, and HDA-3 together with HDA-1 in a SIN3L complex.

### SIN3L and SIN3S complexes both contribute to germline maintenance

To investigate whether any of the SIN-3 interactors we identified are also required for fertility, we measured brood sizes in the corresponding mutants. The SIN3S complex components MRG-1/MRG-15 and HDA-1/HDAC are ubiquitously expressed, well-characterized proteins found in additional chromatin complexes and required for fertility and larval development, respectively (Hajduskova et al., 2018; Bleuyard et al., 2017; Iwamori et al., 2016; Smith et al., 2013; Dufourcq et al., 2002; Passannante et al., 2010; Whetstine et al., 2005). Their role in the germline was therefore not further analyzed here. We instead focused on one other SIN3S component, ATHP-1/PHF12, and the SIN3L complex components HDA-3 and SUDS-3. *athp-1(tm4223)* and *hda-3(ok1991)* are previously described loss-of-function alleles (Beurton et al., 2019; Kawamura and Maruyama, 2020), whereas *suds-3(syb2212)* is a full-deletion allele constructed by CRISPR-Cas9 for this study*.* As previously reported, *athp-1* mutants laid significantly fewer progeny than did wild type (Beurton et al., 2019) (Fig. 2B). A smaller but significant reduction in brood size was also observed in *hda-3* and *athp-1* mutants, whereas *suds-3(syb2212)* mutant animals were fully fertile (Fig. 2B). These results correlate with minor morphological defects observed in both *hda-3* and *athp-1* mutant germlines (Fig. S3). Because SUDS-3 is a core component of the SIN3L complex and is required for its HDAC activity (Alland et al., 2002; Lechner et al., 2000), the absence of an obvious germline phenotype in the corresponding mutant suggests that this complex does not play a prominent role in the maintenance of fertility. ATHP-1 is instead an accessory factor in SIN3S complexes, knockdown of which may negatively impact SIN3S activity in the germline (Xie et al., 2012; Jelinic et al., 2011; Li et al., 2007). Interestingly, simultaneous inactivation of SIN3S and SIN3L complex subunits in both *athp-1;suds-3* and *athp-1;hda-3* double-mutant animals resulted in fully penetrant sterility (Fig. 2B). This genetic interaction suggests common roles in the maintenance of germline function. Alternatively, or in addition, *athp-1*, *hda-3* and *suds-3* may play redundant, *sin-3*-independent functions essential for fertility (Barnes et al., 2018), as suggested by the more severe phenotype of double-mutant animals (no progeny) compared with the *sin-3* null allele (few occasional progeny) (Figs 1C and 2B).

### SIN-3 and HDA-3 have both unique and common regulatory functions in germline gene expression

**Fig. 3.**
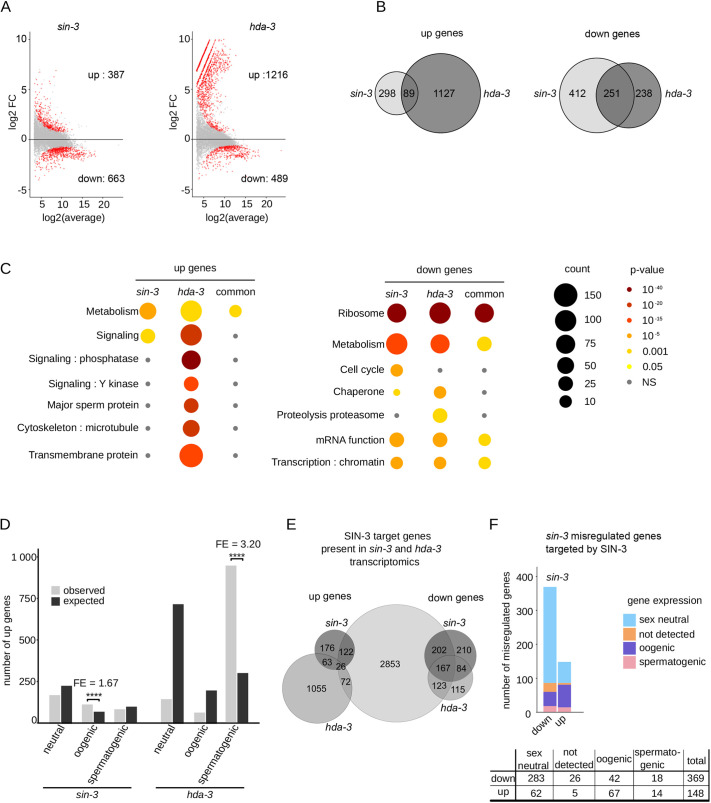
**Transcriptomic analysis of *sin-3* and *hda-3* mutant germlines.** (A) MA plots showing the log2(fold change) of gene expression in *sin-3(syb2172)* mutants (left) and *hda-3(ok1991)* mutants (right) compared with wild type as a function of log2(basemean expression level). *sin-3(syb2172)* animals are M−Z−. Genes that are significantly misregulated in each mutant are in red and were identified using DESeq2 (with FDR<0.05). (B) Venn diagrams showing the overlap between upregulated (left) and downregulated (right) genes in *sin-3* and *hda-3* mutants. (C) WormCat visualization of categories enriched in genes upregulated (left) or downregulated (right) in *sin-3* mutants, *hda-3* mutants or common to both (common). The legend for bubble charts is indicated on the right, with size referring to the number of genes in each category and color referring to the *P*-value. NS, not significant. (D) Enrichment analysis. Bar plot representing the expected (black) and observed (gray) number of upregulated genes in *sin-3* or *hda-3* mutants present in gene sets derived from transcriptomics of adult oogenic or spermatogenic gonads (Ortiz et al., 2014). Enrichment was calculated with hypergeometric tests performed in R. ***P<10^−8^. FE, fold enrichment. Gene numbers in each set are as follows: total number of protein-coding genes 10,063 (5950 sex-­neutral, 1660 oogenic, 2453 spermatogenic); number of oogenic genes upregulated in *sin-3* (108) and in *hda-3* (68); number of spermatogenic genes upregulated in *sin-3* (79) and in *hda-3* (944); number of sex-neutral upregulated genes in *sin-3* (173) and in *hda-3* (149). (E) Venn diagram showing the overlap between SIN-3 ChIP peaks on promoters (Beurton et al., 2019) and up- or downregulated genes in *sin-3* mutant germlines. (F) Bar plot representing the repartition of SIN-3 target genes that are up- or downregulated in the different gene sets defined by Ortiz et al. (2014) in *sin-3* transcriptomics.

To explore how the germline transcriptome is affected by loss of SIN-3, we performed transcriptome profiling on dissected gonads from *sin-3(syb2172)* and *sin-3(tm1276)* young adults. We also included *hda-3(ok1991)* mutant germlines in our analysis because HDA-3 is poorly characterized and found in SIN3L but not SIN3S; its transcription profiling could therefore provide useful insights into SIN3L complex function (Fig. 3). Using DESeq2 (FDR<0.05) to derive lists of differentially expressed genes, we found a significant number of both up- and downregulated genes for *sin-3(syb2172)* compared with wild type (387 and 663, respectively) (Fig. 3A, Table S2), in agreement with SIN-3 acting in both gene repression and activation, as in other systems (Saha et al., 2016; Saunders et al., 2017; Yao et al., 2017; van Oevelen et al., 2008; Gajan et al., 2016). Significantly more genes were found to be misregulated in *sin-3(tm1276)* mutants (2306 up and 2967 down), although there was a large degree of overlap for both up- and downregulated genes (Fig. S4, Table S2). The difference in the total number of misregulated genes may be attributed to differences in sample preparation and processing, or the specific allele used. All subsequent analyses were carried out on genes misregulated in *sin-3(syb2172)* germlines.

In contrast to *sin-3* mutants, in *hda-3* mutants 70% of de-regulated genes were upregulated (1216 out of 1705 total), and of these only 89 were also upregulated in *sin-3(syb2172)* (Fig. 3B). Therefore, as observed for other HDACs (Kadosh and Struhl, 1997; Rundlett et al., 1998; Taunton et al., 1996), *hda-3* acts mainly as a transcriptional repressor, and this function is mostly independent of *sin-3*. Surprisingly, instead, over 50% of *hda-3* downregulated genes were also downregulated in *sin-3(syb2172)* (251 out of 489 total) (Fig. 3B)*.* These may represent direct targets of SIN-3/HDAC-mediated activation (Wang et al., 2009; Chou et al., 2011; Greer et al., 2015; Kim et al., 2013; Kelly and Cowley, 2013), or indirect targets. RT-qPCR analysis confirmed the differential expression of misregulated genes in *sin-3* mutant animals compared with wild type (Fig. S5A). Additional RT-qPCR analysis on *hda-3*, *suds-3* and *athp-1* mutants revealed that three out of five selected genes downregulated in *sin-3* and *hda-3* were also downregulated in *suds-3* mutants, but their de-regulation varied in *athp-1* mutants. For upregulated genes instead, no consistent pattern was observed, although *hda-3* and *suds-3* expression profiles were very similar. This limited analysis is consistent with common de-regulation in SIN3L complex mutants.

Enrichment analysis using WormCat (Holdorf et al., 2020) revealed that among downregulated genes distinct sets of metabolic genes were over-represented in *sin-3(syb2172)* and *hda-3* datasets, and cell cycle genes in the *sin-3(syb2172)* dataset (Fig. 3C). SIN3 is associated with metabolic functions and cell cycle regulation in different species (Chaubal and Pile, 2018), suggesting possibly conserved regulatory functions. Components of GLP-1/Notch signaling and FBF proteins, key regulators of progenitor germ proliferation (Hubbard and Schedl, 2019), were not found in our set of misregulated genes, but ribosomal protein genes were commonly downregulated in both mutants. *hda-3* upregulated genes were enriched in major sperm proteins and tau-tubulin kinases, which are both enriched in spermatogenic gonads. Comparison of our lists with those obtained by transcriptomic analyses of adult oogenic or spermatogenic gonads (Ortiz et al., 2014) confirmed that ‘spermatogenic’ genes are overexpressed in *hda-3* mutants, and revealed that ‘oogenic’ genes are overexpressed in *sin-3(syb2172)* mutants (Fig. 3D).

Our expression analysis does not distinguish direct from indirect targets of SIN-3, and many of the identified changes in gene expression may be an indirect result of SIN-3 loss. To identify potential direct targets of SIN3 regulation, we compared our list of misregulated genes in *sin-3(syb2172)* mutants with a published list of SIN-3 binding sites obtained by chromatin immunoprecipitation with sequencing (ChIP-seq) (Beurton et al., 2019). When considering all misexpressed genes, SIN-3 binding was observed on the promoter of both up- and downregulated genes, with a bias towards downregulated genes (Fig. 3E). Similar analysis revealed that a majority of *hda-3* downregulated genes are bound by SIN-3 (290/489), and, of these, a fraction is commonly downregulated in *sin-3* (167/290): these likely represent targets of a SIN-3/HDA-3 complex*,* consistent with a role for deacetylation in gene activation (Gryder et al., 2019). As expected, the large majority of genes upregulated in *hda-3* were not associated with SIN-3 binding, in agreement with their regulation being independent of *sin-3* (Fig. 3B). Of the set of SIN-3 target genes upregulated in *sin-3(syb2172)* mutants (148), near 50% were oogenic (Fig. 3F). smiFISH analysis revealed that expression of one oogenic gene, the argonaute *vsra-1*, occurred prematurely in *sin-3* mutant germlines, with transcripts detected in the distal region of the gonad where they are not detected in wild type (Fig. S5B).

### SIN-3 contributes to silencing of the X chromosome in the germline

**Fig. 4.**
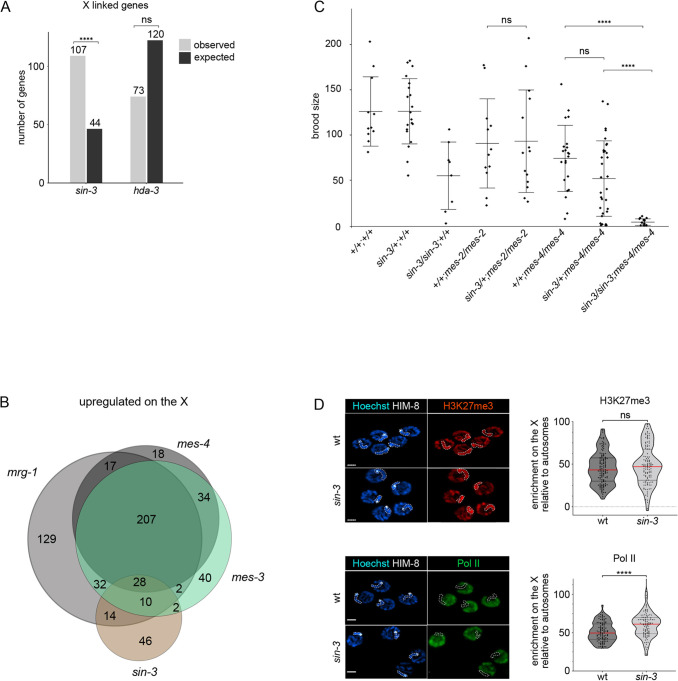
**SIN-3 contributes to X chromosome silencing in the germline.** (A) Enrichment analysis for upregulated genes in *sin-3(syb2172)* and *hda-3(ok1991)* mutants. Bar plot illustrates the expected (black) and observed (gray) number of upregulated X-linked genes in each mutant background. Enrichment was calculated with hypergeometric tests performed in R. ***P<0.0001. ns, not significant. Gene numbers in each set are as follows: total number of analyzed genes for *sin-3* (7757), total number of genes on X chromosome (886), total number of *sin-3* upregulated genes (405), number of *sin-3* upregulated genes on X chromosome (109), total number of analyzed genes for *hda-3* (8407), total number of genes on X chromosome (831), total number of *hda-3* upregulated genes (1238), number of *hda-3* upregulated genes on X chromosome (74). (B) Venn diagram showing the overlap between X-linked genes upregulated in *sin-3(syb2172)*, *mrg-1(qa6200)*, *mes-3(bn199)* and *mes-4(bn173)* mutants. *mrg-1* and *mes* gene lists are from Cockrum and Strome (2022). (C) Loss of *sin-3* enhances *mes-4* sterility. The total number of F1 progeny from animals with the indicated genotype was scored. All homozygote *mes* and *sin-3* mutants are M+Z−. Multiple comparisons were carried out using a generalized linear model with a quasi-Poisson law. ****P<0.0001. Error bars represent mean±s.d. (D) Representative confocal microscopy images of H3K27me3, Pol II and HIM-8 immunolabeling on pachytene-stage nuclei in wild-type and *sin-3(syb2172)* animals. HIM-8 immunolabeling identifies the X chromosome (outlined by dashed lines). Scale bars: 2 µm. Violin plots display the enrichment on the X chromosome relative to autosomes for either H3K27me3 or Pol II immunolabeling. ****P<0.001, one-tailed, unpaired *t*-test). ns, not significant.

Further analysis revealed that a large number (107) of genes upregulated in *sin-3(syb2172)* mutants reside on the X chromosome (Fig. 4A), which represents a significant enrichment and suggests a role for SIN-3 in the repression of X-linked transcripts (Kelly et al., 2002). Because oogenic genes are enriched on the X (Ortiz et al., 2014), we investigated whether their over-representation in the set of *sin-3* upregulated genes reflects upregulation of the X chromosome, or a general de-regulation of the oogenic program. We observed the expected bias for oogenic genes on the X chromosome, but this was not larger than expected (Table S3), and upregulated X-linked genes were not uniquely oogenic. This suggests that de-repression of the X, rather than a general de-regulation of the oogenic program per se, most likely accounts for the enrichment of this class of genes in the set of *sin-3* upregulated genes.

In *C. elegans*, the X chromosomes in XX hermaphrodites and XO males are globally ‘silenced’ during most stages of *C. elegans* germ cell development through the combined activity of the maternal effect sterile (MES) proteins. MES-2/3/6 are homologs of PRC2 components and deposit repressive H3K27me3. MES-4 instead deposits the activating mark H3K36me3 on germline-expressed autosomal genes and antagonizes H3K27me3, leading to its concentration on the X chromosome (Gaydos et al., 2014; Bender et al., 2006; Snel et al., 2022). Previous studies have shown that the large majority of *mes-3* and *mes-4* targets on the X chromosome overlap, consistent with their cooperation in silencing the X chromosome (Cockrum and Strome, 2022). MRG-1, a component of several chromatin complexes in addition to SIN3S (Chen et al., 2010; Huang et al., 2017; Smith et al., 2013; Yochum and Ayer, 2002; Baytek et al., 2022), also contributes to X silencing in the *C. elegans* germline (Takasaki et al., 2007). Comparison of our list of *sin-3(syb2172)* upregulated genes on the X chromosome with a list of MES-3, MES-4 and MRG-1 targets on the X chromosome (Cockrum and Strome, 2022) revealed commonly upregulated genes in the four mutants (28), as well as a larger set genes uniquely in common between *mes-3*, *mes-4* and *mrg-1*, as previously described (Cockrum and Strome, 2022) (Fig. 4B and Table S3 for hypergeometric tests). In addition, 46 genes were upregulated in *sin-3* mutants only: these may be unique targets of SIN3 on the X chromosome (Fig. 4B). We also observed enrichment of X-linked genes among the small set of genes commonly upregulated in *hda-3* and *sin-3* mutants (Fig. S6A), suggesting that SIN-3 may also repress a small set of genes in the context of SIN3L. One of these commonly upregulated genes is *lin-15B*, encoding a THAP domain transcription factor also upregulated in *mes* mutants. Loss of *lin-15B* in *mes-4* mutant animals was shown to reduce X misexpression and prevent germline death (Cockrum and Strome, 2022). We found that *lin-15B(RNAi)* had no effect on the fertility of *sin-3* mutants (Fig. S6B), and its upregulation in *hda-3* mutants is not associated with any obvious germline defects (Fig. S3), suggesting that additional mechanisms contribute to sterility in the absence of *sin-3*.

### Loss of *sin-3* enhances sterility of *mes-2*/PRC2

Altogether, our data show that the absence of *sin-3* results in maternal-effect sterility, reduction in progenitor cell proliferation, and de-repression of X-linked genes. Although less severe, these phenotypes are reminiscent of those found in *mes* mutants (Capowski et al., 1991), and suggest that *sin-3* and *mes* genes may genetically interact. *mes* mutants are fully penetrant, maternal-effect sterile: homozygous *mes/mes* mutant offspring from heterozygous *mes/+* parents are fertile as a result of maternal rescue, but their offspring (M−Z−) are sterile. We compared the fertility of *+/+; mes-2/mes-2* and *sin-3/+; mes-2/mes-2* animals derived from *sin-3(syb2172)/+; mes-2(bn11)/+* mothers. As expected, *+/+; mes-2/mes-2* animals that inherited maternal MES-2 protein were fertile (Fig. 4C) (Capowski et al., 1991), as were *sin-3/+; mes-2/mes-2* animals. *sin-3/sin-3; mes-2/mes-2* animals with maternal SIN-3 and MES-2 contribution were not recovered, suggesting zygotic unviability (McManus et al., 2021). We also compared the fertility of +/+*; mes-4/mes-4*, *sin-3*/+*; mes-4/mes-4* and *sin-3/sin-3; mes-4/mes-4* mutants*.* We found that whereas *+/+; mes-4/mes-4* and *sin-3/+; mes-4/mes-*4 mutants were fertile, *sin-3/sin-3; mes-4/mes-4* animals, which inherit both maternal SIN-3 and MES-4 proteins, were nonetheless sterile, consistent with SIN-3 and MES-4 acting in parallel pathways to promote fertility, most likely through the silencing of distinct subsets of X-linked genes (Fig. 4C).

MES proteins silence the X chromosome by concentrating repressive H3K27me3 on this chromosome (Bender et al., 2006). To test whether de-repression of the X chromosome in *sin-3* mutants and enhancement of the *mes-2* phenotype in the absence of *sin-3* is accompanied by a decrease in H3K27me3 on the X chromosome, we carried out immunolabeling experiments with H3K27me3-specific antibodies on pachytene nuclei, when the X chromosome is normally silenced (Fig. 4D). Using HIM-8 immunolabeling to identify the silenced X chromosome (Phillips et al., 2005; Rappaport et al., 2021), we observed that in wild-type H3K27me3 was enriched on this chromosome with respect to autosomes, as expected (Kelly et al., 2002), and that this enrichment was still observed in *sin-3(syb2172)* mutants. Immunolabeling of the active form of Polymerase II (Pol II) instead revealed a slight but reproducible increase on the X chromosome in *sin-3* mutant germlines, consistent with increased gene expression from this chromosome. Interestingly, in both H3K27me3 and Pol II immunolabeling experiments we observed a small subpopulation of nuclei with decreased H3K27me3 or increased Pol II signal in *sin-3* mutants compared with wild type (Fig. 4D). Our experimental setup did not allow us to establish whether these are the same nuclei; that is, whether nuclei with decreased H3K27me3 also show increased Pol II immunolabeling. Nonetheless, these results suggest that within a single *sin-3* mutant germline, the chromatin and transcriptional state of the X chromosome may vary between individual pachytene nuclei.

### SIN-3 inactivation results in precocious and stochastic transcription of X-linked genes

**Fig. 5.**
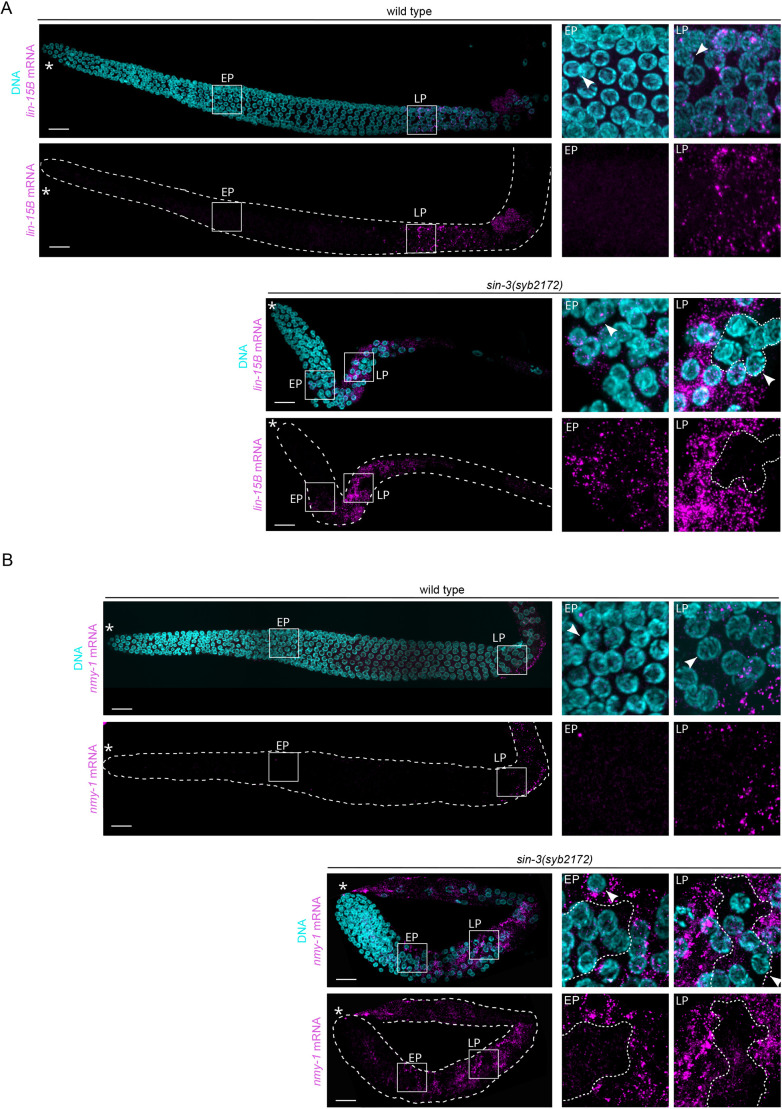
***sin-3* germ nuclei precociously and stochastically de-silence X-linked genes.** (A,B) Images show expression of *lin-15B* (A) and *nmy-1* (B) X-linked transcripts detected by smiFISH analysis in wild-type and *sin-3(syb2172)* germlines. Transcripts are detected as bright nuclear or cytoplasmic dots (magenta). Enlarged views of the boxed regions representing nuclei from early and late pachytene (EP and LP, respectively) are presented on the right. Early and late pachytene nuclei are identified by the typical ‘bowl of spaghetti’ appearance observed by Hoechst staining (cyan), and their position in the germline (Crittenden et al., 2017). White dashed lines outline examples of group of nuclei with few or no transcripts neighboring nuclei with strong expression. Asterisks indicate the distal end of the gonad. Arrowheads indicate examples of nuclei used to identify early and late pachytene regions, based on DAPI morphology. Images are maximum *z*-projections. Scale bars: 10 µm.

To gain further insight into how loss of *sin-3* affects transcription at the single-cell level, we performed smiFISH experiments (Tsanov et al., 2016) with exonic probes for the X-linked genes *lin-15B* and *nmy-1*, which are both upregulated in *sin-3(syb2172)* mutant germlines (Table S2). In wild type, both transcripts were mostly detected as spots starting in late pachytene, consistent with the re-expression of X-linked genes at this stage (Kelly et al., 2002; Tzur et al., 2018) (Fig. 5, Fig. S7). The few spots observed more distally in early/mid pachytene likely represent the few transcriptional events that take place at earlier stages (Fig. 5B). In *sin-3(syb2172)* mutant germlines, by contrast, numerous spots were already detected in nuclei starting in the transition zone/early pachytene, based on DAPI morphology (Fig. 5). Closer examination of smiFISH images (Fig. 5, right panels) showed that in wild type spots are mostly nuclear and perinuclear, whereas in *sin-3* mutant germlines transcripts accumulate in the cytoplasm. Unexpectedly, for both *lin-15B* and *nmy-1* we observed groups of nuclei with few or no spots surrounded by nuclei with many spots (Fig. 5, Fig. S7). This patchy distribution of transcripts in *sin-3* mutants suggests re-expression of X-linked transcripts occurs in a stochastic manner in single nuclei.

### Increased acetylation on specific residues in *sin-3* mutants

**Fig. 6.**
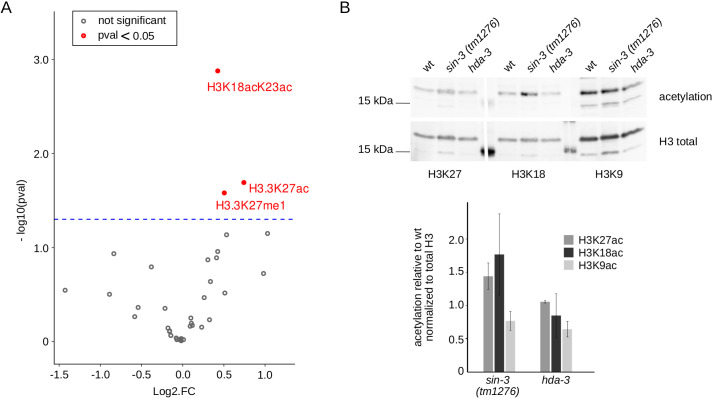
**Specific residues in *sin-3(tm1276)* mutants are more acetylated than in wild type.** (A) Volcano plot representing the log2 fold change (log2FC) on H2, H3 and H4 peptide modification abundances in *sin-3(tm1276)* versus wild-type young adult worms plotted against statistical significance [−log10(*P*-value)]. Modifications that are significantly different between both genotypes are plotted as red dots. Horizontal blue dashed line represents a significant threshold of *P*<0.05. (B) Semi-quantitative western blot analysis of acetylation levels in total extracts from wild-type, *sin-3(tm1276)* and *hda-3(ok1991)* young adults. Acetylation of H3K9, H3K18 and H3K27 was detected by fluorescence after western blotting using specific antibodies. Levels of each modification were quantified using ImageJ and normalized to total H3 levels. Bar plot shows the mean of two independent biological replicates; error bars correspond to s.d.

To identify histone modifications potentially targeted by SIN-3 and its associated HDACs, we carried out MS analysis of purified histones from *sin-3(tm1276)* young adults, a stage at which the germline is fully developed. We chose the *tm1276* allele because the sterility of *syb2172* does not allow the culturing of a large enough number of animals required for this type of analysis. Although the abundance of most quantified histone acetylations was not significantly altered in *sin-3(tm1276)* mutants, we detected a significant increase in the bivalent mark K18AcK23Ac on histone H3, and in K27Ac and K27me1 on histone H3.3 (Fig. 6A). The abundance of histone H3 with acetylation on either K18 or K23 residues alone was not significantly affected. Co-existence and positive interplay between K18Ac and K23Ac has previously been described (Klein et al., 2019; Schwämmle et al., 2014). Semi-quantitative western-blot analysis using antibodies that detect acetylation marks on both histone H3 and H3.3 showed a slightly increased acetylation on K27 and K18, but not K9, in total extracts from *sin-3(tm1276)* mutant young adults (Fig. 6B). No change in acetylation levels was observed in *hda-3* mutant extracts. By contrast, an increase in H3K18Ac and a smaller increase in H3K27Ac was observed upon auxin-induced depletion of HDA-1 (Fig. S8A), suggesting that HDA-1 is responsible for deacetylation of these residues. ChIP-qPCR analysis on a set of genes misregulated in both *sin-3* mutant alleles showed no clear correlation between acetylation levels and mRNA levels: we observed both increased and decreased H3K27Ac on the promoter of genes upregulated in *sin-3* mutants (Fig. S8B). This lack of correlation may reflect heterogeneity in the population of germ cells, as indicated by the smiFISH analysis. Alternatively, changes in these marks may occur elsewhere in the genome and their detection will require genome-wide approaches. Increased H3K27Ac in the germline was further confirmed in immunolabeling experiments on *sin-3(tm1276)* animals using H3K27Ac antibodies (Fig. S8C).

## DISCUSSION

In this study, we have shown that the highly conserved transcriptional co-regulator SIN3 is essential for fertility in *C. elegans*. We biochemically identified SIN-3 interactors that differentially contribute to fertility and provide data suggesting that specific class I HDACs may reside in distinct complexes (Fig. S9). We further uncovered a role for SIN-3 in the timely repression of X-linked genes, and showed by single-cell analysis that re-expression of X-linked genes in *sin-3* mutant germlines occurs prematurely and in a stochastic manner. These results suggest that the absence of SIN-3 may not uniformly affect gene expression in all germ cells.

Maternal-effect sterility associated with *sin-3* inactivation was revealed here using a CRISPR-Cas9 null allele, *syb2172*, and was missed in previous studies using the hypomorphic allele *sin-3(tm1276)* (Choy et al., 2007; Pandey et al., 2018; Sharma et al., 2018; Beurton et al., 2019). Sterility in *sin-3(syb2172)* is associated with reduced proliferation of progenitor germ cells, a process tightly controlled by GLP-1/Notch signaling and RNA regulators (Hubbard and Schedl, 2019). However, expression of these factors is not affected in *sin-3* mutant germlines, nor did we find any evidence for defects in the switch between self-renewal and differentiation that could account for a reduced pool of progenitor cells (Crittenden et al., 2017). What we instead observed is increased expression of X-linked genes. Silencing of the X chromosome in the germline is under the control of MES proteins; in *mes* mutants, upregulation of X-linked genes results in death of nascent germ cells, a more severe phenotype than observed in the absence of *sin-3* (Bender et al., 2006; Gaydos et al., 2014). This may be partly accounted for by the significantly smaller number of SIN-3 target genes on the X chromosome. A crucial target for repression by MES proteins on the X chromosome is the *lin-15B* THAP domain transcription factor, and its inactivation prevents germline death in *mes* mutants (Cockrum and Strome, 2022). Although *lin-15B* is also upregulated in both *sin-3* and *hda-3* mutant germlines, its inactivation in *sin-3* mutants had no effect on fertility, suggesting that in this context its upregulation alone does not contribute to sterility. Alternatively, LIN-15B protein abundance may only increase in *mes*, but not in *sin-3* or *hda-3* mutants. Consistent with additional SIN-3 functions in the germline, transcriptomics analysis showed that SIN-3 regulates genes with roles in metabolism and cell cycle regulation. Additional roles for SIN-3, independent of MES, either on the X chromosome itself or at other loci, are supported by genetic analysis in which we observed that loss of *sin-3* enhances the sterility of *mes-4* M+Z− mutants with maternal MES-4 contribution. Loss of SIN-3 may also influence germline gene expression and overall chromatin organization, as suggested by altered distribution of repressive H3K9me2 in *sin-3(tm1276)* mutant animals (Checchi and Engebrecht, 2011; She et al., 2009).

Our analysis also provided important insight into the composition and function of distinct SIN3-associated complexes in a single tissue. We confirm previous results showing that knockdown of the SIN3S complex subunit ATHP-1/PHF12 results in reduced fertility (Beurton et al., 2019), and show that animals lacking SUDS-3, an essential component of SIN3L complexes (Banks et al., 2020; Clark et al., 2015), are instead fully fertile. Like SIN-3, the SIN3S component MRG-1 is required for silencing of the X chromosome and fertility (Takasaki et al., 2007), and both SIN-3 and MRG-1 are required for piRNA silencing in the germline (Kim et al., 2021). These shared phenotypes are consistent with common functions as part of the same complex, although MRG-1 most likely has additional functions alone or in the context of additional complexes (Baytek et al., 2022; Hajduskova et al., 2018; Bleuyard et al., 2017; Iwamori et al., 2016; Smith et al., 2013). Although SIN3L subunits alone may not be required for fertility, the observation that *athp-1; hda-3* and *athp-1; suds-3* double mutants are fully sterile suggests at least partial redundancy of the two complexes, or additional functions for the encoded subunits independent of SIN-3.

Interestingly, whereas mammalian HDAC1 and HDAC2 are mostly considered to be functionally redundant, we found that both HDA-3 and HDA-1 are present in the SIN3L complex identified in this study, whereas SIN3S components only co-purify with HDA-1 (Baytek et al., 2022; Beurton et al., 2019). HDA-1 has been found in at least two additional complexes, NuRD and MEC (Passannante et al., 2010; Kim et al., 2021), but an HDA-3-containing complex has not been described to date. Mammalian HDAC1 and -2 co-purify in mammalian SIN3 complexes (Banks et al., 2018, 2020), but also have unique functions and partners (Terzi Cizmecioglu et al., 2020; Quaas et al., 2022). Notably, in mouse embryonic stem cells ARID4B was found to interact with SIN3A and HDAC1, but not HDAC2 (Terzi Cizmecioglu et al., 2020). Interestingly, the mammalian counterpart of the transcription factor TCF-19, identified here as an accessory component of SIN3L, was found to interact with NuRD/HDAC at the promoter of gluconeogenic genes (Sen et al., 2017), suggesting that it may play a role in the recruitment of distinct HDAC-containing complexes to chromatin. Consistent with unique functions, *Hdac1* knockout mice are embryonic lethal (Lagger, 2002), whereas an *Hdac2* deletion is viable (Trivedi et al., 2007). Similarly, *hda-1* inactivation results in larval lethality, whereas *hda-3* mutant are viable (Kawamura and Maruyama, 2020 and this work).

Our expression profiling shows that, as in other systems, loss of SIN-3 results in both repression and activation of gene expression (Saha et al., 2016; Saunders et al., 2017; Yao et al., 2017; van Oevelen et al., 2010), consistent with the presence of HDACs at promoters of both actively transcribed and repressed genes (Wang et al., 2009). SIN-3 may influence gene expression in several ways. On the silent X chromosome, we did not detect depletion of H3K27me3 in the absence of SIN-3, arguing against a simple model in which de-silencing results from loss of repressive chromatin. Nonetheless, loss of SIN-3 may result in alterations in the H3K27me3 landscape too small to be detected by immunofluorescence. Immunolabeling of *sin-3* mutants did show a small increase in Pol II on the X chromosome in pachytene, and smiFISH analysis revealed that X-linked transcripts accumulate stochastically in early meiotic cells, when the X chromosome is normally silenced (Kelly et al., 2002). Together, these results suggest that SIN-3 may not elicit a uniform response on its targets on the X chromosome, but instead act as an ‘epigenetic switch’ at individual target genes, resulting in ON or OFF transcriptional states. This may occur in a concerted manner, or independently at each locus. Perturbation of lysine acetylation is one mechanism that could potentially modify the frequency of transcriptional bursting, or switching between states (Nicolas et al., 2018; Tunnacliffe and Chubb, 2020), but whether SIN3 influences gene expression at specific loci through HDAC activity remains to be established. We identified H3K18AcK23Ac as a SIN-3-dependent bivalent mark, and confirmed increased H3K18Ac in total extracts from *sin-3(tm1276)* mutant animals. Although a partially reconstituted SIN3B complex displayed very low deacetylase activity at every H3 acetyl-Lys site tested in *in vitro* assays on reconstituted nucleosomes, a slight preference for H3K18Ac, H3K23Ac and H3K27Ac compared with K9Ac was observed (Wang et al., 2020). Preferential binding to di-acetyladed residues has been reported for several readers of acetylation (Parthun, 2012; Barman et al., 2021; Obi et al., 2020), with one of the two bromodomains of BRDT showing highest affinity for H3K18AcK23Ac (Miller et al., 2016).

Although ChIP-qPCR experiments of hand-picked genes did not reveal a correlation between increased H3K27Ac and expression levels in *sin-3* mutants, stochastic effects of SIN-3 depletion on gene expression, as we observed on the X chromosome, may require single-cell approaches to address this question further. In addition, SIN-3 may play non-canonical roles in repressive chromatin (Torres-Campana et al., 2022), target non-histone substrates (Milazzo et al., 2020), or have functions independent of HDAC catalytic activity, including nucleosome assembly (Chen et al., 2012), as suggested by the physical interaction with the NAP1 chaperone (Moshkin et al., 2009 and our data). Some of these activities may also contribute to SIN-3-mediated gene activation. Future studies in simple systems, such as the *C. elegans* germline, may help gain insight into common regulatory mechanisms involving SIN3 and aid in the identification and study of pathogenic SIN3 variants associated with disease.

## MATERIALS AND METHODS

### Strains and maintenance

Nematode strain maintenance was as described previously (Brenner, 1974). Wild-type N2 (Bristol) was used as reference. Strains used are as follows [asterisk indicates from Beurton et al. (2019); double asterisk indicates created for this study]: *PFR590, *sin-3(tm1276)* I; **PHX2172, *sin-3(syb2172)/hT2[bli-4(e937) let-?(q782) qIs48] I*; *PFR588, *cfp-1(tm6369)* IV; *PFR593, *athp-1(tm4223) III*; **PHX2212, *suds-3(syb2212)* V; *PFR740, *hda-3(ok1991) I*; **PFR746, *athp-1(tm4223)*III; *hda-3(ok1991)*I; **PFR747, *athp-1(tm4223)* III; *suds-3(syb2212)*V; **PHX4769, *arid-1(syb4769)* V; SS186, *mes-2(bn11)*; *unc-4(e120)/mnC1[dpy-10(e128) unc-52(e444)]* II. CRISPR-Cas9 alleles were created by SunyBiotech using the primers listed in Table S4. A file displaying the sequence of wild-type, *suds-3* and *sin-3* alleles, and the junction of the deletion in CRISPR-Cas9 alleles, is available on GitHub (https://gitbio.ens-lyon.fr/cbedet/supplemental_files_cbedet/). PHX2172 animals were maintained by picking wild-type fluorescent worms (pharyngeal GFP), which are heterozygous, and segregating wild-type fluorescent animals, arrested hT2 aneuploids, and GFP-negative *sin-3(syb2172)* homozygotes.

### Genetic crosses

Double mutants, transgenes and CRISPR-Cas9 knock-in alleles were obtained by crossing. Males used for crosses were either heterozygotes obtained from crossing wild-type males with hermaphrodites carrying the mutation or transgene of interest, or homozygotes obtained by heat-shock of mutant L4 hermaphrodites. Single hermaphrodites from crosses were isolated and allowed to lay eggs overnight, then genotyped by PCR and/or screened for the desired phenotype. This was repeated until double or triple homozygotes were obtained.

### Scoring brood size

Ten L4 worms per genotype were isolated onto single NGM plates seeded with OP50 bacteria, allowed to grow into egg-laying adults overnight at 20°C, then transferred every 12 h to fresh plates until they ceased laying eggs. For each plate, the number of progeny was scored 24 h after removal of the mother, and the total brood size per animal calculated.

### Observation of live worms

Worms were placed on 2% agarose pads in a 15 µl drop of 10 mM levamisole in M9. Slides were examined using either brightfield, fluorescence or differential interference contrast microscopy with a Zeiss Axio Imager A2 microscope or Nikon AZ100 M Zoom microscope.

### Immunofluorescence microscopy on dissected gonads

Experiments were as previously described (Herbette et al., 2017). Briefly, young adults (L4+12 h) were transferred to an empty plate for 15 min to remove bacteria, then transferred to a drop of dissection buffer (M9 0.4×, levamisole 10 mM) on poly-lysine-coated slides. Worms were cut at the pharynx and gonads extracted using 30g ½″ needles (BD Microlance), fixed in 3.2% paraformaldehyde (Thermo Fisher Scientific) for 5 min, and placed on dry ice prior to freeze-cracking (Strome and Wood, 1982). Slides were immersed in methanol at −20°C for 1 min, washed three times in 1×PBS (Sigma-Aldrich) with 0.1% Tween 20 (Sigma-Aldrich), blocked for 40 min in 0.1% Tween 20 in 1× PBS (PBST) and 1% bovine serum albumin (MP Biomedicals), and incubated with primary antibodies overnight at 4°C in a humid chamber. Slides were washed three times in PBST, incubated with secondary antibodies for 50 min, then washed for 7 min in PBST plus 5 μg/ml Hoechst 33342 (Sigma-Aldrich), twice, 10 min each, in PBST, and mounted in mounting medium (90% glycerol and 0.4% propyl gallate in 1× PBS). All antibodies were diluted in 0.1% Tween 20 and 1% bovine serum albumin in 1× PBS. Antibodies used were: mouse anti-H3K37me3 (61017, Active Motif, 1:300), rabbit anti-H3K27ac (39133, Active Motif, 1:5000), mouse anti-H3 (14269 Cell Signaling Technology, 1:500), rat anti-RNA Pol II (04-1571, Millipore, 1:1000), rabbit anti-pH3 (SC-8656-R, Santa Cruz Biotechnology, 1:100), rabbit anti-HIM-3 (kind gift from Monique Zetka, McGill University, Montreal, Canada), rat anti-HIM-8 (kind gift from Abby Dernburg, UC Berkeley, USA), goat anti-rat IgG (A-21434, Invitrogen, 1:1000), goat anti-mouse Alexa Fluor Plus 555 (A32727, Invitrogen, 1:1000), goat anti-rabbit Alexa Fluor Plus 647 (A32733, Invitrogen, 1:1000). Antibodies were diluted in PBST. Images were acquired with either a Zeiss LSM980 confocal microscope or a Yokogawa CQ1 spinning disk confocal microscope.

### Genetic crosses between *sin-3* and *mes* mutants

*mes-2(bn11)*/+ animals were crossed with *sin-3(syb2172)/+* males. *bn11*/*bn11* M-Z- animals are sterile owing to a fully penetrant failure in germ-cell proliferation (Capowski et al., 1991). Because of the absence of genetic markers closely linked to *bn11*, we could not easily genotype *mes-2* worms. Instead, we inferred genotypes based on the phenotypes (sterility and presence or complete absence of germ cells). We focused on animals derived from self-fertilization of *sin-3*/+*; mes-2*/+ animals that produced sterile progeny, and individually PCR genotyped these for the presence of *sin-3(syb2172)*. *sin-3(+)/sin-3(+)* and *sin-3(syb2172)/sin-3(+)* animals that produce sterile progeny are *mes-2(bn11)*/*mes-2(bn11)*, and we used a binocular loupe to verify the absence of germ cells in the gonad and confirm the *mes* phenotype. We examined the gonads of sterile progeny of *sin-3(syb2172)*/*sin-3(syb2172)* animals to identify *mes-2(+)/mes-2(+)* animals (100% sterile progeny with germ cells in their gonad); *mes-2(bn11)/mes-2(+)* animals (mix of sterile animals with or without germ cells in their gonads); and *mes-2(bn11)/mes-2(bn11)* animals (100% sterile progeny with no germ cells in their gonads). We did not recover *sin-3(syb2172)*/*sin-3(syb2172); mes-2(bn11)*/*mes-2(bn11)* animals from more than 200 animals scored, suggesting that zygotes with this phenotype are unviable. For crosses with *mes-4*, we mated *dpy-11(e224) mes-4(bn23) unc-4(e76)/+* animals with *sin-3(syb2172)/+ males*. Progeny were genotyped based on the DpyUnc phenotype for *mes-4* and by PCR for *sin-3*. For brood size scoring, parents were singled as L4 larvae on NGM plates, and allowed to reach adulthood and lay eggs for 4 days at 20°C. Brood size was counted 2 days after parent elimination.

### Measuring signal ratio between X chromosomes and autosomes

Immunofluorescent acquisitions of late-pachytene nuclei obtained with a Zeiss LSM980 confocal microscope were analyzed using Fiji. Maximum projections of four to six stacks of 0.16 µm were obtained, and nuclei in which both an X chromosome (identified using HIM-8 immunolabeling) and at least one autosome were present in the same transversal position were analyzed in subsequent steps. The average intensity on the X chromosome and one autosome was quantified, background intensity subtracted and the ratio between the X chromosome and autosome was then quantified on each nucleus analyzed. One-hundred nuclei were analyzed per genotype. Statistical analysis was performed using one-tailed, unpaired *t*-tests.

### Acridine Orange staining of apoptotic cells

Staining of apoptotic cells was as previously described (Gumienny et al., 1999). In brief, 1 ml of 50 µg/ml Acridine Orange (AO; Sigma-Aldrich) in 1× M9 was dropped onto plates containing 20-30 young adults (L4+12 h). Plates were kept at 20°C for 2 h in the dark, and worms were then transferred onto fresh plates to wash off any excess AO. Worms were individually transferred onto agarose pads and imaged using a Zeiss LSM980 confocal microscope. Images were processed using Fiji (Schindelin et al., 2012), and cells positives for AO staining were counted in a *z*-stack. Twenty gonads per genotype were counted.

### Immunoprecipitation experiments

Immunoprecipitations were performed on frozen embryos prepared by hypochlorite treatment from animals grown at 20°C on enriched NGM seeded with 10× concentrated HB101 bacteria (Beurton et al., 2019). Embryos were washed once in IP buffer (50 mM HEPES/KOH, pH 7.5; 300 mM KCl; 1 mM EDTA; 1 mM MgCl_2_; 0.2% Igepal-CA630; and 10% glycerol) and flash-frozen in beads in liquid nitrogen. Embryos were then ground to powder, resuspended in one bead volume of IP buffer containing 2× complete protease inhibitors (Roche) and sonicated on ice at an amplitude of 30% for 2.5 min (15 s ON/15 s OFF pulses) using an Ultrasonic Processor (Bioblock Scientific). Protein extracts were recovered in the supernatant after centrifugation at 20,000 ***g*** for 15 min at 4°C. Protein concentration was estimated using the Bradford assay (Bio-Rad Protein Assay Dye). All immunoprecipitations were performed with 70 mg of total protein extract in 10 ml diluted in IP buffer. Each sample was incubated for preclearing with a 200 μl slurry of binding-control magnetic agarose beads (ChromoTek, bmab) for 1 h at 4°C. Then, 200 μl of GFP-TRAP MA (ChromoTek, gtma) or 300 μl RFP-TRAP MA beads slurry (ChromoTek, rtma) were added to the sample. Bead incubation was performed 3 h on a rotator at 4°C. Beads were collected with a magnet, washed three times in IP buffer and once in Benzo buffer (HEPES/KOH 50 mM, pH 7.5; KCl 150 mM; EDTA 1 mM; MgCl_2_ 1 mM; Igepal-CA630 0.2%; and glycerol 10%). Beads were then incubated in 400 μl of Benzo buffer containing 2500 units of benzonase (Sigma-Aldrich) for 1 h at 4°C and washed three times in IP buffer. Eluates were recovered by incubation at 95°C for 10 min in 60 μl of 1× LDS buffer (Thermo Fisher Scientific). One-tenth of each eluate was resolved on a 4-12% NuPage Novex gel (Thermo Fisher Scientific) and stained with SilverQuest staining kit (Thermo Fisher Scientific), then 40 μl of the eluates was analyzed by MS.

### MS-based proteomic analyses of IP eluates

Proteins from IP eluates solubilized in Laemmli buffer were stacked in the top of a 4-12% NuPAGE gel (Invitrogen), stained with Coomassie blue R-250 (Bio-Rad) before in-gel digestion using modified trypsin (Promega, sequencing grade) as previously described (Casabona et al., 2013). The resulting peptides were analyzed by online nanoliquid chromatography coupled to MS/MS (Ultimate 3000 RSLCnano and Q-Exactive HF, Thermo Fisher Scientific) using 120 min and 90 min acetonitrile gradients for SIN-3 and ARID-1 interactomes, respectively. For this purpose, the peptides were sampled on a precolumn (300 μm×5 mm PepMap C18, Thermo Fisher Scientific) and separated in a 75 μm×250 mm C18 column (Reprosil-Pur 120 C18-AQ, 1.9 μm, Dr. Maisch). The MS and MS/MS data were acquired using Xcalibur 4.0 (Thermo Fisher Scientific).

Peptides and proteins were identified by Mascot (version 2.8.0, Matrix Science) through concomitant searches against the Uniprot database (*Caenorhabditis elegans* taxonomy, 20220531 download), a homemade database containing the sequences of the bait proteins, and a homemade database containing the sequences of classical contaminant proteins found in proteomic analyses (e.g. human keratins, trypsin). Trypsin/P was chosen as the enzyme and two missed cleavages were allowed. Precursor and fragment mass error tolerances were set at, respectively, 10 and 20 ppm. Peptide modifications allowed during the search were: Carbamidomethyl (C, fixed), Acetyl (Protein N-term, variable) and Oxidation (M, variable). Proline software (Bouyssié et al., 2020; version 2.2.0) was used for the compilation, grouping and filtering of the results: conservation of rank 1 peptides, peptide length≥6 amino acids, FDR of peptide-spectrum-match identifications <1% (Couté et al., 2020), and a minimum of one specific peptide per identified protein group. Proline was then used to perform a spectral counts-based comparison of the protein groups identified in the different samples. Proteins from the contaminant database were discarded from the final list of identified proteins. To be considered as a potential binding partner of a bait, a protein must be detected with a minimum of three specific spectral counts, identified only in the positive eluate, or enriched at least three times in this eluate compared with the corresponding control eluate on the basis of spectral counts.

### RNA sequencing and data analysis

For each biological replicate of wild type, *sin-3(syb2172)* mutants and *hda-3(ok1991)* mutants, gonads from 9-12 young adults were dissected in UltraPure water on microscope slides as described for immunofluorescence, except that the entire dissection protocol was performed at 4°C to immobilize worms without use of anesthetic. RNA was isolated, reverse-transcribed, amplified and cleaned up following the Smart-Seq2 protocol described by Serra et al. (2018). Three independent biological replicates were performed for each strain. Libraries were generated at the GenomEast Platform (IGBMC, Strasbourg, France) using the SMART-Seq v4 UltraLow Input RNA kit (Clontech) followed by the Nextera XT DNA sample preparation Kit (Illumina) and sequenced using the Illumina Hiseq 4000 technology (1×50 bases). For wild type and *sin-3(tm1276)* mutants, gonad dissections, RNA extractions and sequencing of wild type and *sin-3(tm1276)* young adult worms were performed exactly as described by Herbette et al. (2020).

For data analysis, fastq files were processed with fastp (version 0.20.1), reads were mapped to the *C. elegans* reference genome (WS278) by RNA-STAR (version 2.7.3a). and gene expression level in each sample was calculated by htseq-count (version 0.7.2). Differential expression between each mutant strain and wild type was then calculated with DESeq2 (version 1.36.0) using a homemade R script (R version 4.2.2; available on https://gitbio.ens-lyon.fr/cbedet/supplemental_files_cbedet). At this step, principal components analysis revealed that one replicate for *hda-3* mutant was an outlier and thus it was discarded for further analysis. Of the 46,934 annotated genes, only protein-coding genes with a baseMean>10 were selected in each condition (8208 genes for *hda-3* and 7555 for *sin-3*).

### RNAi feeding experiments

*sin-3* homozygous M−Z− animals derived from M+Z− mothers raised on *lin-15B(RNAi)* were transferred to fresh RNAi feeding or empty vector control plates at the L4 stage and allowed to develop to adults. F1 progeny were individually repicked on fresh RNAi plates and scored for fertility. The efficacy of *lin-15B(RNAi)* was measured by placing *lin-38(n751)* animals on RNAi plates and scoring the Muv (Multivulva) phenotype resulting from simultaneous inactivation of synMuvA (*lin-38*) and synMuvB (*lin-15B*) genes (Fay and Han, 2000).

### RNA extraction and RT qPCR analysis from whole worms

Worms were collected in M9 and washed three times with M9 and once with UltraPure distilled water (Invitrogen). Pellets were resuspended in two volumes of TRI Reagent (MRC, Inc.) and 0.2 volume of chloroform (Sigma-Aldrich) was added for every volume of TRI Reagent added. Samples were incubated at room temperature for 5 min and centrifuged for 5 min at 12,000 ***g***. The supernatant was collected, and an equal volume of absolute ethanol (Sigma-Aldrich) was added. RNA was extracted using RNeasy (QIAGEN) kit following the manufacturer's protocol. RNA was eluted in 30 µl of UltraPure water and the integrity and concentration of RNA was measured with TapeStation 4200 and RNA Screen Tape (Agilent). For each condition, two samples of 500 ng purified RNA were retrotranscribed using the Transcriptor Universal cDNA Master kit (Roche), then pooled together and diluted five times in UltraPure water. qPCR was performed with Takyon SYBR 2× MasterMix (Eurogentec) on a CFX Connect real-time detection system (CFX 96 Bio-Rad). RNA levels were normalized to the mean of *act-1* and *cdc-42* genes. Two technical replicates were performed for each of two to three independent biological samples. For each primer pair, efficiency, linear range and fusion curves were checked using serial dilutions of cDNA.

### mRNA detection by smiFISH

smiFISH was performed using a smiFISH protocol provided by the Hubstenberger lab and adapted from Tsanov et al. (2016). Primary probes were designed using the R script Oligostan (https://bitbucket.org/muellerflorian/fish_quant/src/master/Oligostan/; Tsanov et al., 2016). *lin-15B* and *nmy-1* primary probes were designed with rules 1, 2, 3, 4 and 5 in the PNASfilterOption and *vrsa-1* primary probes were designed with rules 1, 2, 4 and 5. For all probes, MinGC content was set up at 0.4 and maxGC content at 0.6. Primary probes sequences are provided in Table S5. Probes were produced by hybridization of primary probes with a FLAPx-Cy5 secondary probe in Tris EDTA 1× complemented with 100 mM NaCl. Denaturation was performed at 95°C for 5 min and the mix was allowed to cool down at room temperature until it reached 35-40°C. Worms were dissected for gonad isolation on poly-lysine-coated slides in PB Buffer (Na_2_HPO_4_ 80 mM, NaH_2_PO_4_ 4mM) complemented with 100 mM levamisole. Gonads were pre-fixed in 4% paraformaldehyde for 4 min at room temperature and immediately frozen on dry ice. After freeze-cracking, gonads were fixed in cold 4% paraformaldehyde for 20 min at room temperature and washed twice with PB buffer before a 20 h fixation at 4°C in cold 70% ethanol. Before hybridization, slides were washed twice in 1× PBS and once in 1× SSC complemented with 15% formamide. An additional 20 min incubation was performed for equilibration in 15% formamide in 1× PBS before proceeding with hybridization. For hybridization, probes were diluted 40 times in Stellaris RNA hybridization buffer complemented with 10% formamide. Samples were incubated for at least 16 h at 37°C. Samples were washed twice with 1× SSC complemented with 25% formamide for 30 min and incubated with Hoechst 33342 for 5 min before two additional washes in 1× PBS. Samples were mounted in ProLong Antifade Mounting media (Invitrogen) and imaged using an AxioObserver Z1 LSM980 confocal microscope with AiryScan2 (Zeiss).

### Histone purification and MS analysis

Histones from wild-type and *sin-3(tm1276)* young adult worms were purified following ‘Basic protocol 2’ published by Millan-Ariño et al. (2020). MS was then performed according to ‘Basic Protocol 3’ and histone peptides were analyzed as described in the same article by bottom-up MS with a *C. elegans*-adapted version of EpiProfile 2.0 software (Yuan et al., 2018). Raw data from EpiProfile were analyzed following recommendations from Thomas et al. (2020; https://github.com/DenuLab/HistoneAnalysisWorkflow). Briefly, raw peptide abundance values were filtered to remove all modifications with missing values in more than one replicate for each genotype. Normalization of each modification was performed by dividing it by the sum of all modifications in the replicate. Finally, normalized values for which the s.d. was more than 60% of its average were discarded. Statistical analysis was realized using the linear model:

lm (normalized peptide abundance∼genotype) with a homemade script in R (available at https://gitbio.ens-lyon.fr/cbedet/supplemental_files_cbedet). All samples were analyzed in biological triplicate. Mean abundance of all peptides in wild type and *sin-3 (tm1276)* is given in Table S6.

### Western blot analysis on histone marks

Wild-type N2, *hda-3(ok1991)* and *sin-3(tm1276)* young adult worms were collected in M9 buffer, washed three times, pelleted and frozen in dry ice. For *sin-3(syb2172)* animals, 25 homozygous non fluorescent worms (see ‘Strains and maintenance’ section) were hand-picked on 60 mm plates and allowed to grow until no food was available. A total of five plates containing mostly adult worms were collected in M9 buffer, washed three times, pelleted and frozen in dry ice. To generate *hda-1*-depleted worms, animals carrying an auxin-inducible degron allele of *hda-1* (Kim et al., 2022) were grown on a single 14 cm plate, adult animals bleached and embryos hatched overnight. L1 larvae were then grown either 14 cm plates with or without auxin (1 mM) until the young adult stage. Worms were collected in M9 buffer, washed three times, pelleted and frozen in dry ice. After thawing, pellets were resuspended in TNET buffer [50 mM Tris-HCl, pH 8, 300 mM NaCl, 1 mM EDTA, 0.5% Triton X-100 and cOmplete™ Protease Inhibitor Cocktail (Merck, 11697498001)] and lysed with zirconium beads (Lysing Matrix Y, MP Biomedicals, 116960050) using a Precellys 24 homogenizer (Ozyme) with the following parameters: 6000 rpm 2×10 s. Homogenates were centrifuged at 20,000 ***g*** for 7 min and supernatants aliquoted and frozen at −80°C. Total protein amount was quantified by the Bradford assay (Bio-Rad Protein Assay Dye) and 27 µg of protein extracts were loaded on 12% NuPage Novex gels for western blot analysis. After transfer, membranes were incubated overnight with the following antibodies diluted at 1:2500: anti-H3 (clone 1B1B2, Cell Signaling Technology, 14269), anti-H3K9ac (Active Motif, 39137) anti-H3K27ac (Active Motif, 39133) and anti-H3K18ac antibody (Active Motif, 39755). Membranes were then incubated for 1 h with goat anti-rabbit DyLight™ 800 (Invitrogen, SA5-10036) and IRDye^®^ 680RD goat anti-mouse (LI-COR, 926-68070) diluted at 1:10,000. Image acquisition was performed using a ChemiDoc MP apparatus (Bio-Rad). Quantification was carried out using ImageJ, and each acetylation signal was normalized to the level of histone H3. Two independent biological replicates were used for each strain for quantification.

### ChIP-qPCR analysis on germ nuclei

Germ nuclei from wild-type or *sin-3(tm1276)* animals were prepared using an adapted homemade protocol from Han et al. (2019). See supplementary Materials and Methods for further details.

### Quantification of H3K27 acetylation signal

Maximum intensity projections of whole germlines acquired on Yokogawa CQ1 spinning disk confocal microscope using the same setting for all images, were opened in ImageJ, and ten germlines per strain from two biological replicates were processed identically. Intensities of antibody signal from the distal germline (mitotic and transition zones) or the mid-germline (early and mid-pachytene regions) were measured, normalized to Hoechst 33342 signal, and averaged after the background signal was removed. Statistical analysis was performed using one-tailed, unpaired *t*-tests.

## Supplementary Material

10.1242/develop.201755_sup1 Supplementary information

Table S1. Mass spectrometry-based characterization of SIN-3 and ARID-1 interactomes

Table S2. List of significant misregulated genes in hda-3(ok1991) allele as compared to wild-type

Table S3. Hypergeometric tests related to Figure 4B and to the repartition of sin-3(syb) upregulated oogenic genes on the X chromosome

Table S5. Sequences of smiFISH DNA probes
