# Supplementary material for: SIN-3 acts in distinct complexes to regulate the germline transcriptional program in Caenorhabditis elegans
Source: Development. 2023 Oct 17;150(21):dev201755. doi: 10.1242/dev.201755 (PMC10617626; doi:10.1242/dev.201755)
Supplement: Supplementary information [file develop-150-201755-s1.pdf]

Supplementary Materials and Methods

ChIP-qPCR analysis on germ nuclei

12 x 14 cm enriched plates containing each ~ 200,000 young adult worms were used for the purification of one nuclei pellet. Worms from 6 plates were recovered in M9 buffer and washed at least 3 times with M9 to get rid of bacteria. The pellet was cross-linked with 2% formaldehyde (Thermo Scientific™ #28908) for 28 min at RT on a rotating wheel, quenched with 1M Tris pH=7.4 for 5 min and washed 2X in cold M9 buffer and 1X in cold NPB buffer (NPB: 10 mM HEPES pH 7.6, 10 mM KCl, 1.5 mM $MgCl_2$, 1 mM EGTA, 0.25 M sucrose, 0.025% TritonX-100, 1mM DTT and cOmplete™ Protease Inhibitor Cocktail (Beltran et al., 2021). Cross-linked worms were resuspended in 6 ml of NPB, transferred into a 7 ml prechilled glass Dounce homogenizer (Wheaton, Clearance: 0.05 +/- 0.025mm), and homogenized by performing 4 times 20 tight strokes with a quarter turn after each stroke. Homogenates corresponding to 2 sets of 6 plates were pooled, volume was adjusted to 20 ml with NPB and transferred into a 50 ml canonical tube, vortexed for 30 seconds at medium-high speed and placed on ice for 5-10 min. This step was repeated once. The homogenate was filtered through a 40 µm followed by a 10 µm cell strainer, centrifuged at 2,500 g for 4 min at 4° C, and pelleted nuclei were recovered in 1 ml NPB, transferred to a 1.5 mL DNA-LoBind tube and an aliquot DAPI-stained to check the quality of the preparation. Supernatant was discarded, and the pellet (~ 50-100 µl) frozen in liquid nitrogen or dry ice and kept at -80°C until used for chromatin shearing. For shearing, one nuclei pellet was resuspend into 1 ml of TE + SDS 0,1% , transferred into a Covaris milliTUBE 1 mL with AFA® fiber and sonicated in a S220 Covaris apparatus for 20 min using the following parameters :PIP = 100 W, Duty Factor = 20%, CPB = 200. The homogenate was centrifuged at 10,000 g for 10 minutes at 4°C and the supernatant corresponding to the chromatin extract was checked and quantified on a Tapestation 4200 [Agilent] on a D1000 HS screen tape. Aliquots containing 2-4 ug chromatin were flash frozen in liquid nitrogen and kept at -80°C until used for ChIP. For H3K27ac ChIP experiments on wt or *sin-3(tm1276)* worms, each chromatin extract (2-4 ug per IP) was incubated in 800 µl TSE 150 (20 mM Tris-HCL pH 8.1 ,150 mM NaCl, 2mM EDTA, 1% TritonX-100 and 0.1% SDS) with 2 µl of anti-H3K27ac antibody (Active Motif # 39133) for 4-5h at 4°C (40 µl were kept as Input). 30 µl of protein A Dynabeads (Invitrogen) were added in each sample for an additional 1 h incubation. Beads were washed once for 5 min at RT with 800 µl of the following buffer : TSE 150, TSE 500 (20 mM Tris-HCL pH 8.1 ,500 mM NaCl, 2mM EDTA, 1% TritonX-100 and 0.1% SDS), TSE 1M (20 mM Tris-HCL pH 8.1 ,1 M NaCl, 2mM EDTA, 1% TritonX-100 and 0.1% SDS), TEL (10 mM Tris-HCl, 0.25 M LiCl, 1% IGEPAL-CA630, 1% sodium

Development • Supplementary information

deoxycholate, 1 mM EDTA) and then twice in TE tween20 (10 mM Tris-HCl, 1 mM EDTA, 0.1% tween 20). Beads were resuspended in 100 µl of TE + SDS 1%, incubated for 20 min at 37°C with 0.5 µL of RNase A (10 mg/mL) and then incubated overnight at 65 °C with 2 µL of proteinase K (15–20 mg/mL) with shaking (1100 rpm). Inputs were incubated for 60 min at 37°C with 1 µL of RNase A (10 mg/mL), volume was adjusted to 100 µl with TE + SDS 1% and they were incubated overnight at 65 °C with 2 µL of proteinase K (15–20 mg/mL) with shaking (1100 rpm). Inputs were purified with NucleoSpin Gel and PCR Clean-up Mini kit (Macherey-Nagel # 740609.50 MN05) with NTB buffer according to manufacturer's instructions. Eluates recovered from proteinA beads were purified with NGS NucleoMag beads (Macherey-Nagel # 744970.50) according to manufacturer's instructions. Both inputs and ChIPped DNA were recovered in 40 µl of 10 mM Tris. qPCR on ChIP was performed with Takyon SYBR 2X MasterMix (Eurogentec) on a CFX Connect real-time detection system (CFX 96 Biorad).

Reference

Beltran, T., Pahita, E., Ghosh, S., Lenhard, B. and Sarkies, P. (2021). Integrator is recruited to promoter–proximally paused RNA Pol II to generate Caenorhabditis elegans piRNA precursors. EMBO J. 40, e105564. doi:10.15252/embj.2020105564

Development • Supplementary information

Fig. S1

A

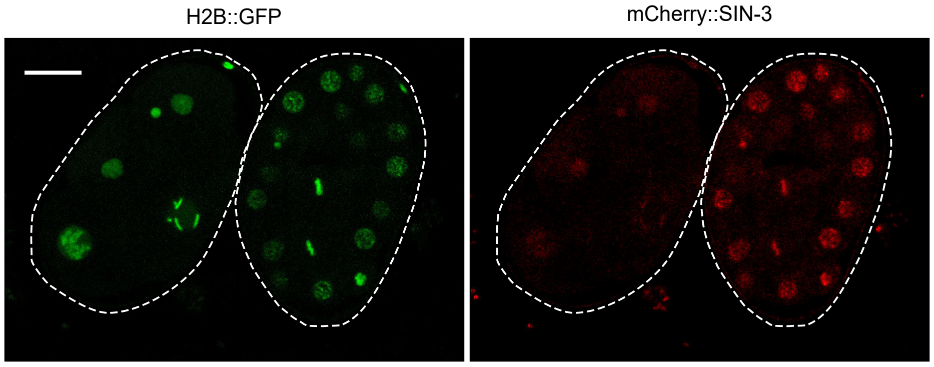

B

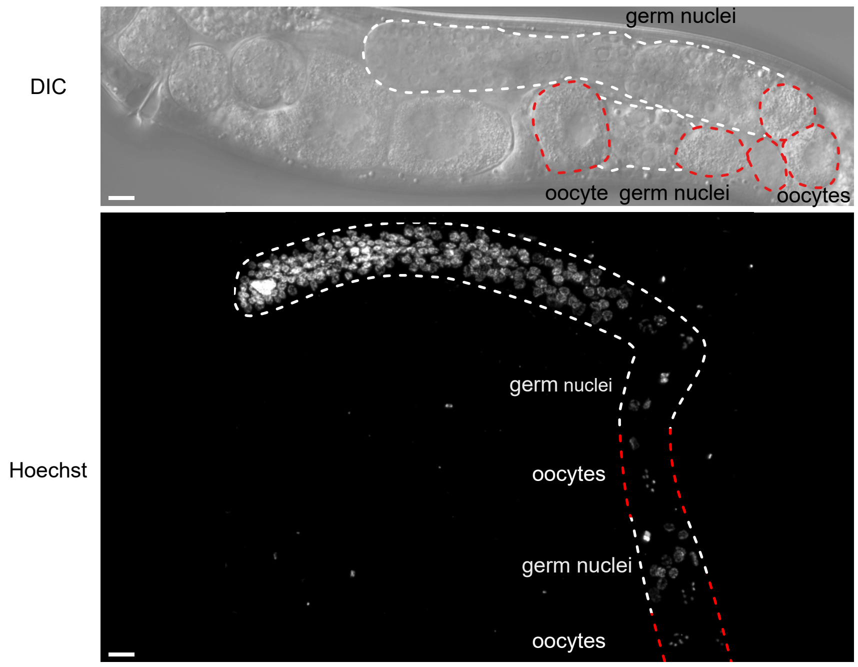

C

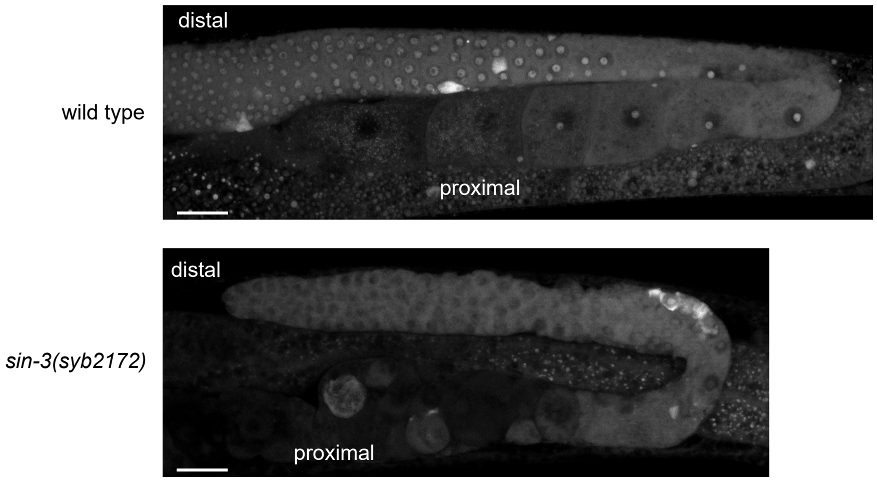

Development • Supplementary information

Fig. S1. SIN-3 localization in embryos and Gogo phenotype of *sin-3(syb2172)* germlines. (A) Representative images of embryos expressing H2B::GFP and mCherry::SIN-3. Scale bar = 10 µm (B) Representative images of germlines displaying the Gogo phenotype by DIC (top panel) and Hoechst-staining of DNA (bottom panel). Germ nuclei and oocytes are outlined respectively with a white and red dotted line in DIC image. Scale bar is 10µm. ((Beurton et al., 2019). (C) Longer exposure of AO staining in Figure 1 showing absence of signal in the proximal gonad. Scale bars = 15 µm

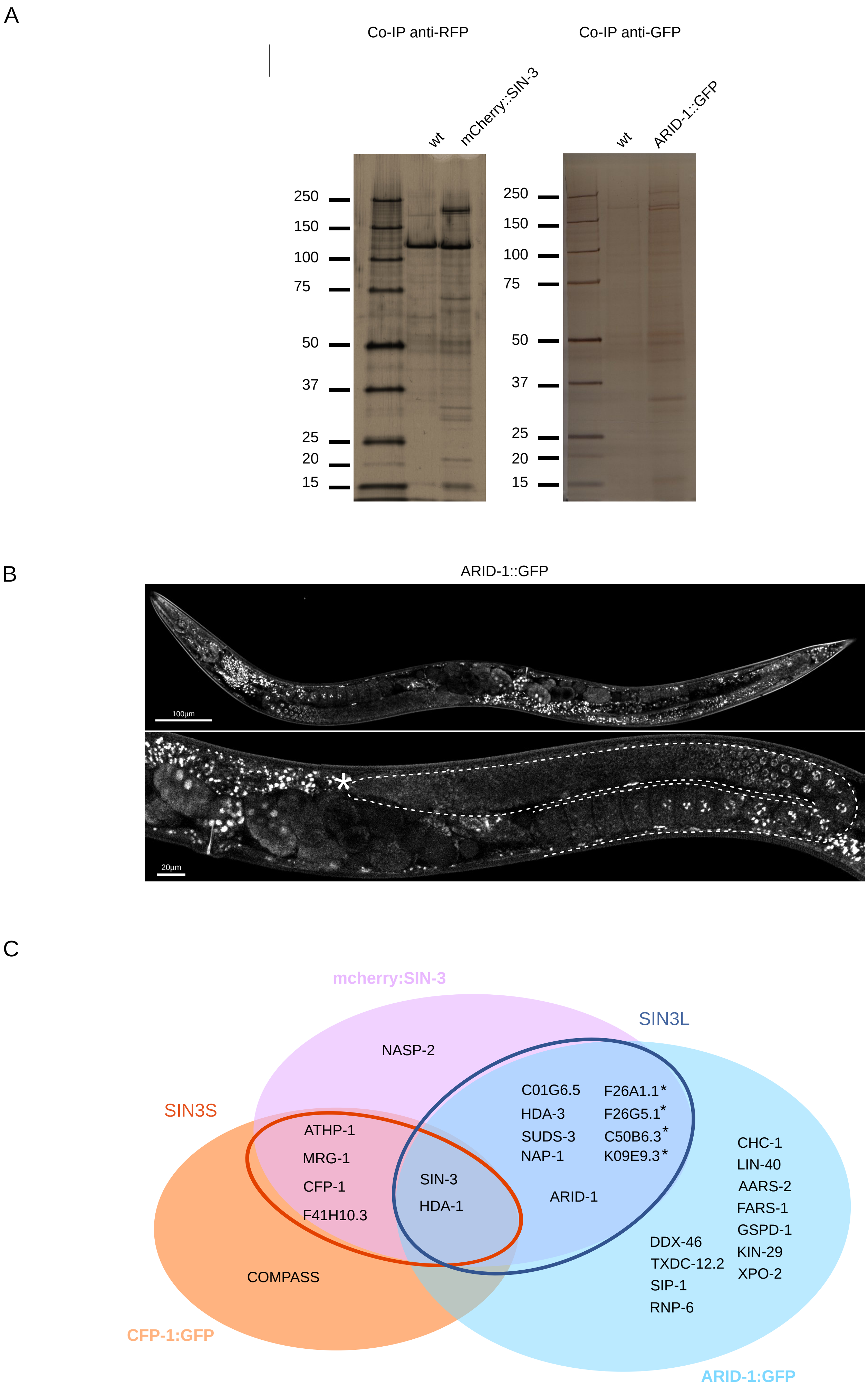
A
Co-IP anti-RFP
Co-IP anti-GFP
wt
mCherry::SIN-3
wt
ARID-1::GFP
250
150
100
75
50
37
25
20
15
B
ARID-1::GFP
100µm
20µm
C
mcherry:SIN-3
SIN3L
SIN3S
CFP-1:GFP
ARID-1:GFP
NASP-2
C01G6.5
HDA-3
SUDS-3
NAP-1
F26A1.1*
F26G5.1*
C50B6.3*
K09E9.3*
ATHP-1
MRG-1
CFP-1
F41H10.3
SIN-3
HDA-1
ARID-1
COMPASS
CHC-1
LIN-40
AARS-2
FARS-1
GSPD-1
KIN-29
XPO-2
DDX-46
TXDC-12.2
SIP-1
RNP-6

Fig. S2. Silver staining of immunoprepitated samples, and *in vivo* localization of ARID-1. (A) Silver staining of mCherry::SIN-3 (left) and ARID::GFP (right) immunoprecipitated samples from 70 mg of *C. elegans* embryonic extracts. 1/10th of the sample was loaded on a 4-12% NuPage gel. (B) Representative confocal microscopy images of whole worms with endogenous ARID-1 tagged by GFP (ARID-1::GFP) using CRISPR-Cas9. Acquisitions were on live anesthetized worms. Germlines are outlined by a dotted line. (*) marks the distal end of the germline. (C) Venn diagram showing overlap in proteins identified by SIN-3 and ARID-1 IP-MS in this study, and previous CFP-1 IP-MS (Beurton et al., 2019). The proteins in this diagram have Specific Spectral Counts (SSC) > 15 and a fold enrichment (FE) > 5 in both SIN-3 and ARID-1 IP-MS (except for HDA-1 : FE = 4,5 for SIN3 and FE = 3,2 for ARID-1 IP-MS). Proteins marked with (*) correspond are *C. elegans*-specific.

Fig. S3

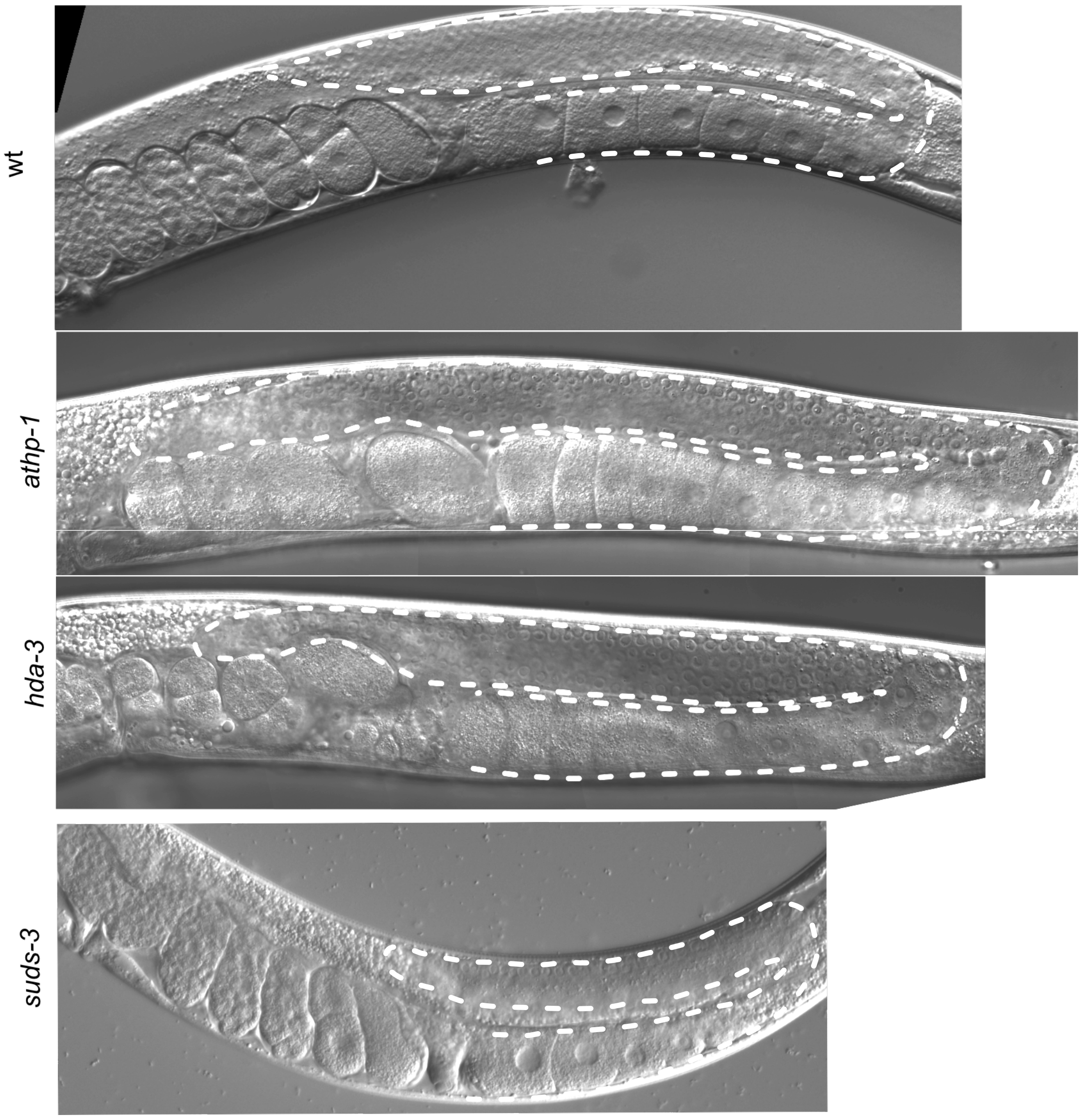

Fig. S3. Nomarski images of mutant *athp-1*, *hda-3* and *suds-3* young adults.

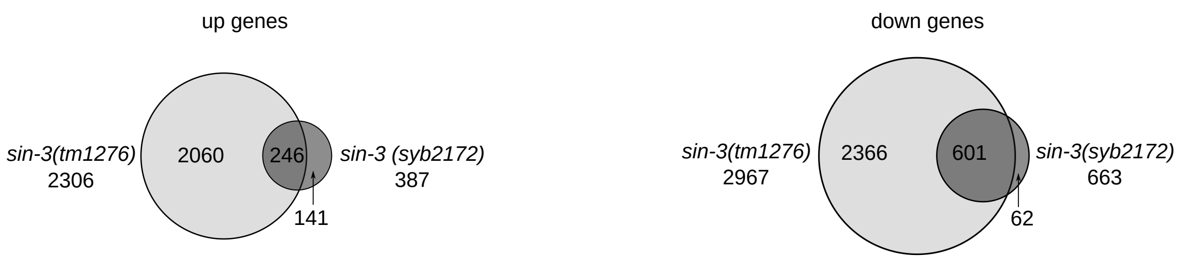

Fig. S4. Commonly regulated genes in *sin-3(tm1276)* and *sin-3(syb2172)* mutant germlines.

Venn diagram shows overlap between upregulated (left) and downregulated (right) genes.

Development • Supplementary information

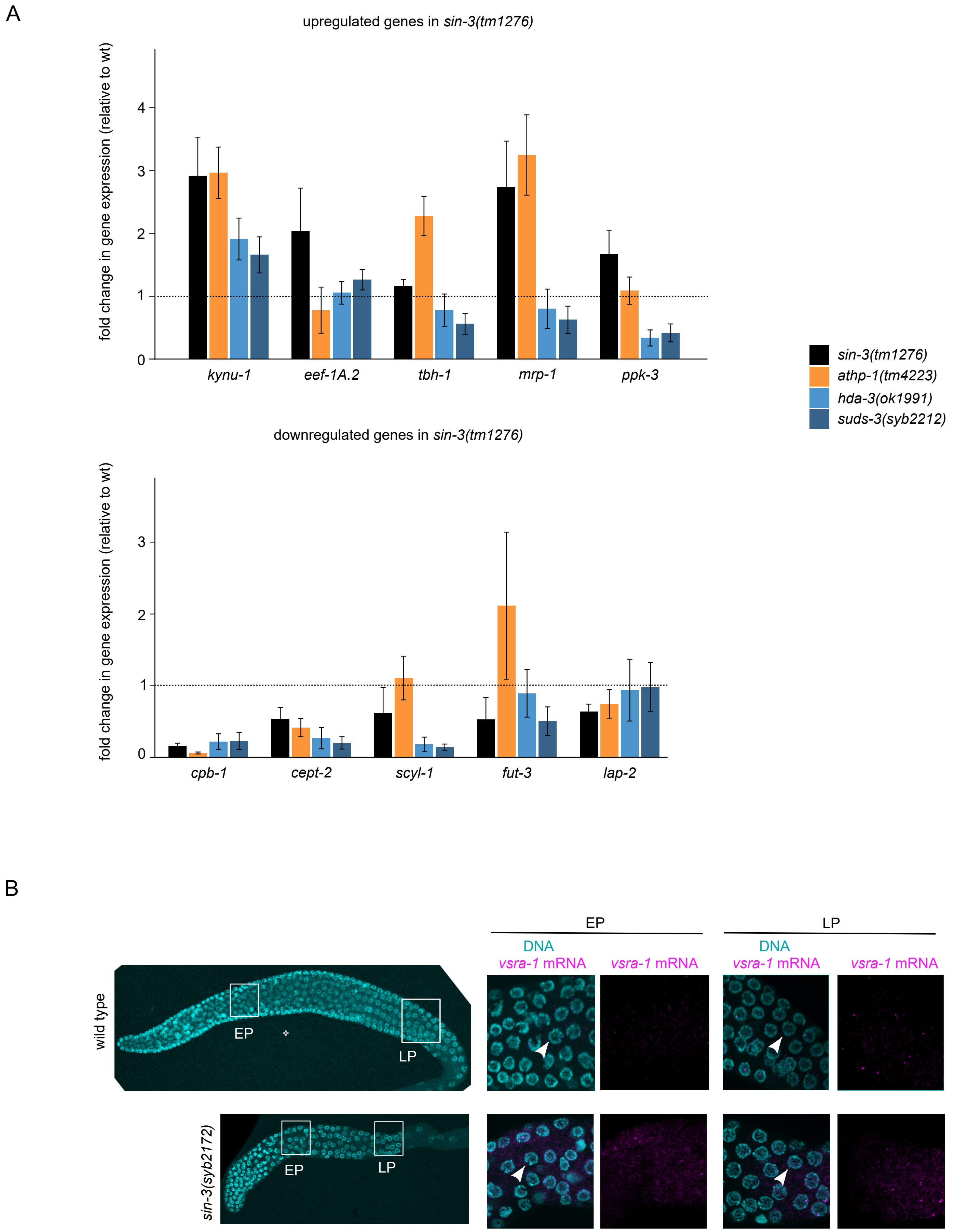

A
upregulated genes in sin-3(tm1276)
fold change in gene expression (relative to wt)
0
1
2
3
4
kynu-1
eef-1A.2
tbh-1
mrp-1
ppk-3
sin-3(tm1276)
athp-1(tm4223)
hda-3(ok1991)
suds-3(syb2212)
downregulated genes in sin-3(tm1276)
fold change in gene expression (relative to wt)
0
1
2
3
cpb-1
cept-2
scyl-1
fut-3
lap-2
B
EP
LP
DNA
vsra-1 mRNA
vsra-1 mRNA
DNA
vsra-1 mRNA
vsra-1 mRNA
wild type
EP
LP
sin-3(syb2172)
EP
LP

Fig. S5. RT-qPCR analysis of misregulated genes in SIN3S and L complex subunit mutants, and smiFISH of representative oogenic gene. (A) RNA was extracted from *sin-3(1276)*, *athp-1(tm4233)*, *hda-3(ok1991)* and *suds-3(syb2212*) young adults. RNA levels were normalized to the mean of *act-1* and *cdc-42* genes. Two technical replicates were performed for each of two to three independent biological samples. (B) smiFISH detection of *vsra-1* transcripts in wildtype and *sin-3*(*syb2172*) gonads. Images of wildtype and mutant germlines (left panels) and zoom-in of boxed region in early (EP) and late (LP) pachytene regions (right panels). Arrows indicate nucleus that were used to identify meiotic regions.

Development • Supplementary information

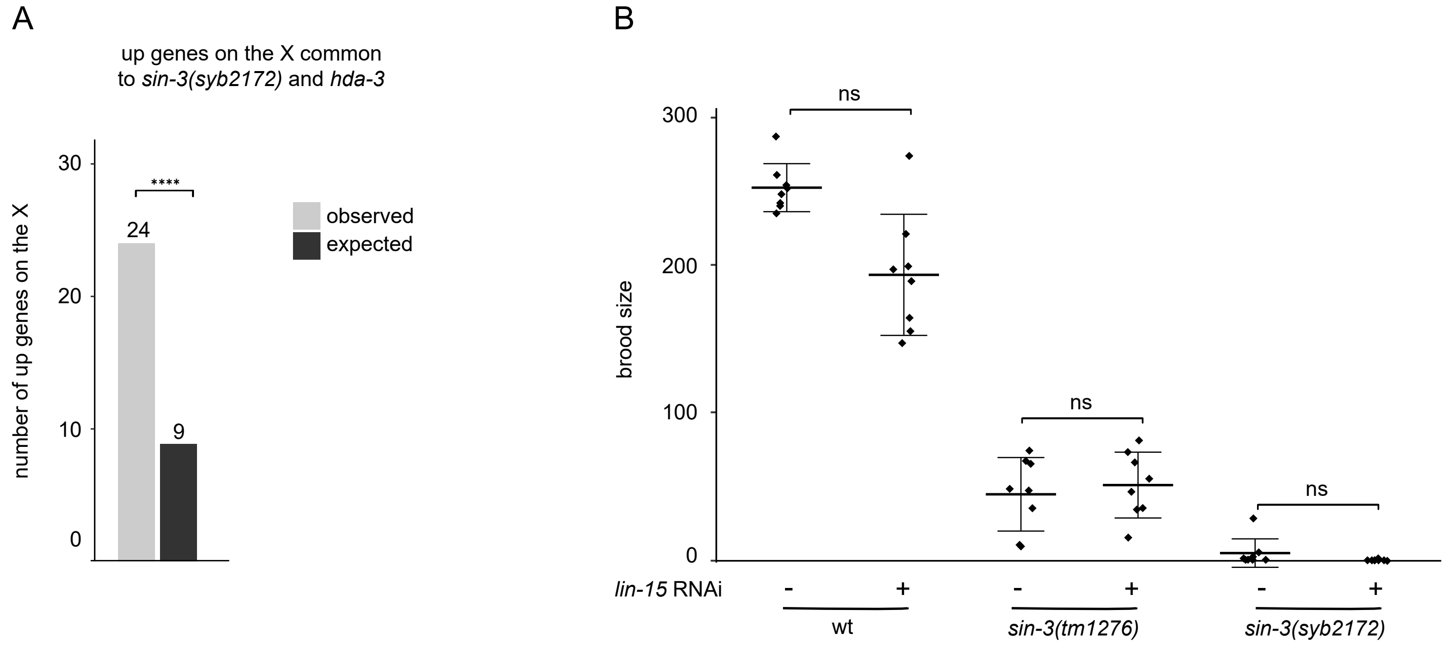

Fig. S6. Enrichment of X-linked genes in list of genes commonly regulated in *sin-3(syb2172)* and *hda-3(ok1991)* mutants, and *lin-15*(RNAi) in *sin-3(syb2172)* mutants. (A) Bar plot showing the expected (black) and observed (gray) number of upregulated genes on the X chromosome common to both mutants. Enrichment was calculated with hypergeometric tests performed in R. *** p-value $<10^{-5}$. (B) Brood size as a read-out of fertility following inactivation of *lin-15B* by RNAi. Statistics were performed using a global linear model with quasi Poisson law (glm (brood_size ~ genotype, family = quasipoisson) Post-hoc pairwise comparison was done using emmeans package in R. ns = not significant.

Development • Supplementary information

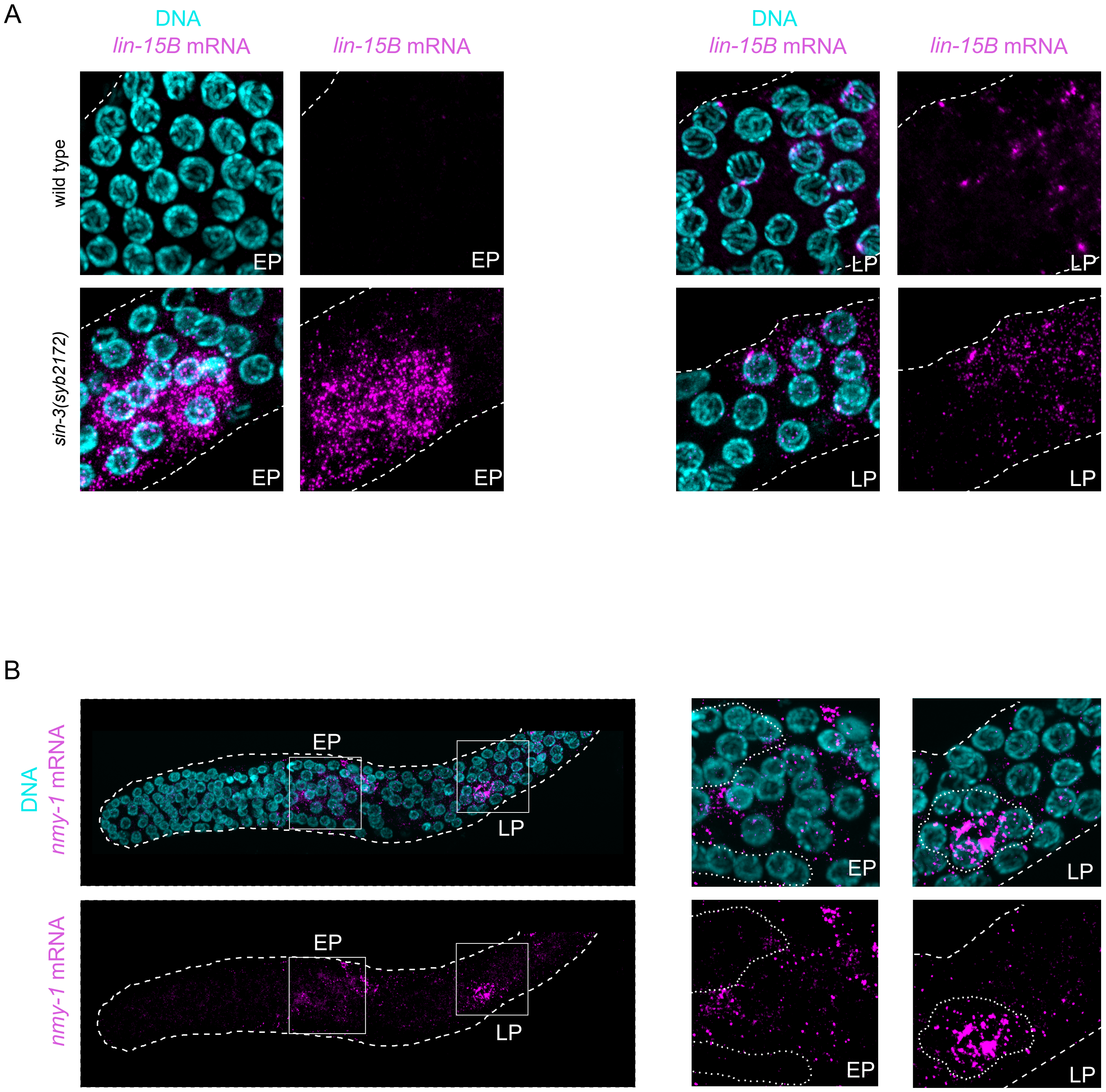

Fig. S7. Additional examples of smiFISH analysis of X-linked gene in wildtype and *sin-3*(*syb2172*) gonads. (A) Expression pattern of *lin-15B* transcripts in wildtype and *sin-3*(*syb2172*) early (EP) and late pachytene (LP) nuclei (identified based on DAPI morphology and position). (B) Expression pattern of *nmy-1* transcripts in a *sin-3*(*syb2172*) gonad. Zoom-in of the transition early pachytene region (EP) and of the late pachytene stage are shown. White broken lines outline examples of group of nuclei with few or no transcripts neighboring nuclei with precocious expression on EP panel and a group a nuclei overexpressing *nmy-1* surrounded by nuclei with a wildtype expression pattern on LP panel. See Figure 5B for *nmy-1* transcript pattern in a wildtype gonad. Scale bar is 20 µm.

Development • Supplementary information

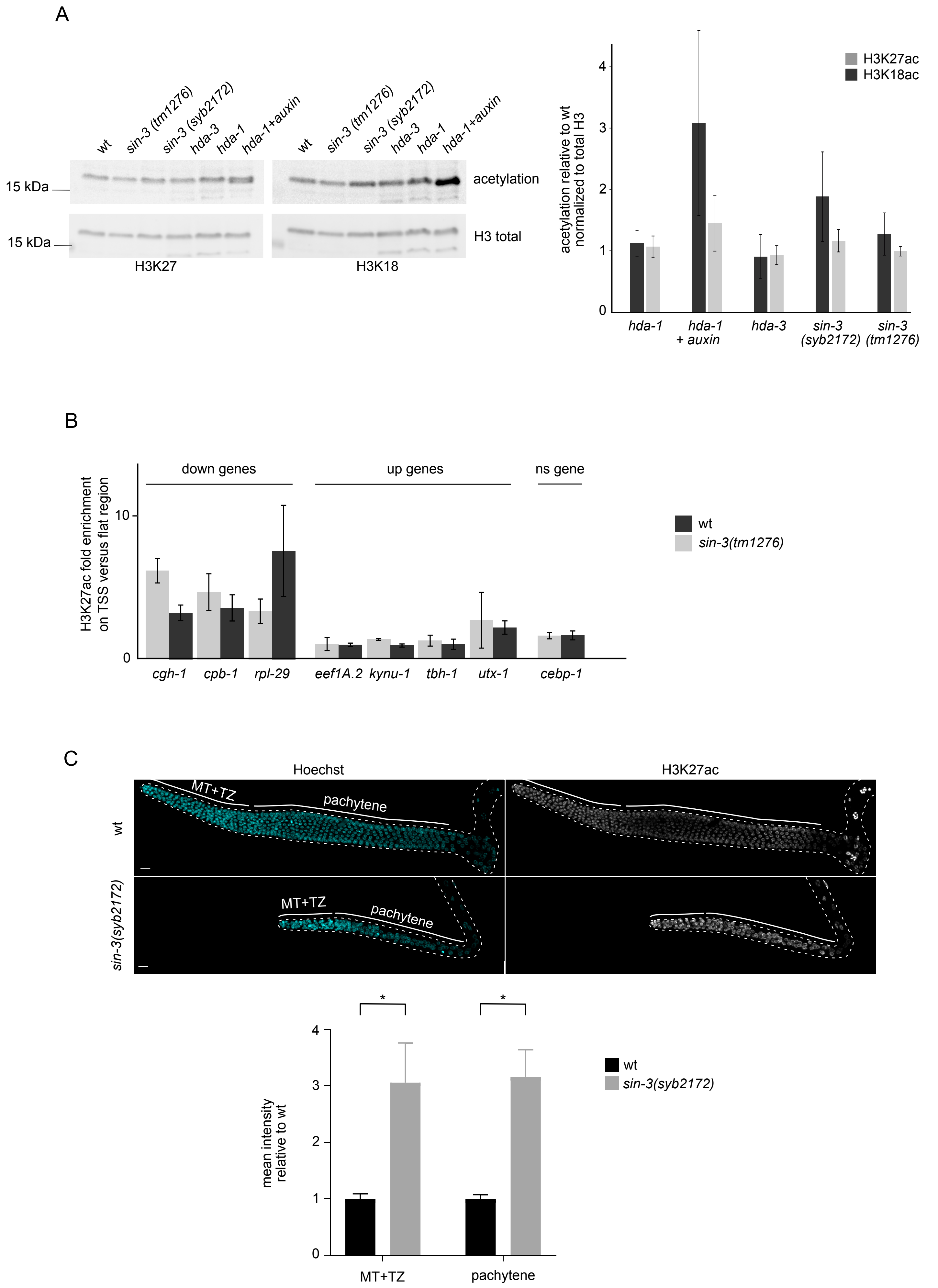

Development • Supplementary information

Fig. S8. Acetylation levels in wildtype and *sin-3* mutants. (A) Semi-quantitative western blot analysis of acetylation levels in total extracts from wildtype, *sin-3(tm1276)*, *sin-3(syb2172)*, *hda-3(ok1991)* and HDA-1 auxin-depleted young adults. Acetylation of H3K18 and H3K27 was detected by fluorescence after western blot using specific antibodies. Levels of each modification were quantified with Image J and normalized to total H3 levels. Barplot represented the mean of two independent biological replicates; error bars correspond to SD. (B) H3K27Ac ChIP qPCR in wildtype and *sin-3(tm1276)* young adults. For each gene tested, two primer pairs were designed: one targeting the TSS of the gene of interest and a second one at least 2 kb downstream from the TSS targeting a region with little or no H3K27ac signal (flat region) in ChIPseq experiments (Han et al., 2019). Barplot represent fold enrichment in H3K27Ac on TSS versus flat region in *sin-3* and wt. Two to four biological replicates were performed. (C) Representative confocal microscopy images of H3K27Ac on dissected germlines from wildtype and *sin-3(syb2172)* animals. Germlines are outlined with a broken line. Images correspond to a Max intensity projection using Fiji. Scale bar, 10µm. Bar graphs show quantification of H3K27ac signal in the mitotic and transition zone (MT+TZ) and pachytene region displayed in the images. Data show changes in the mean pixel intensities relative to wt ± SD. Unpaired Student's t-test * $p < 0.05$.

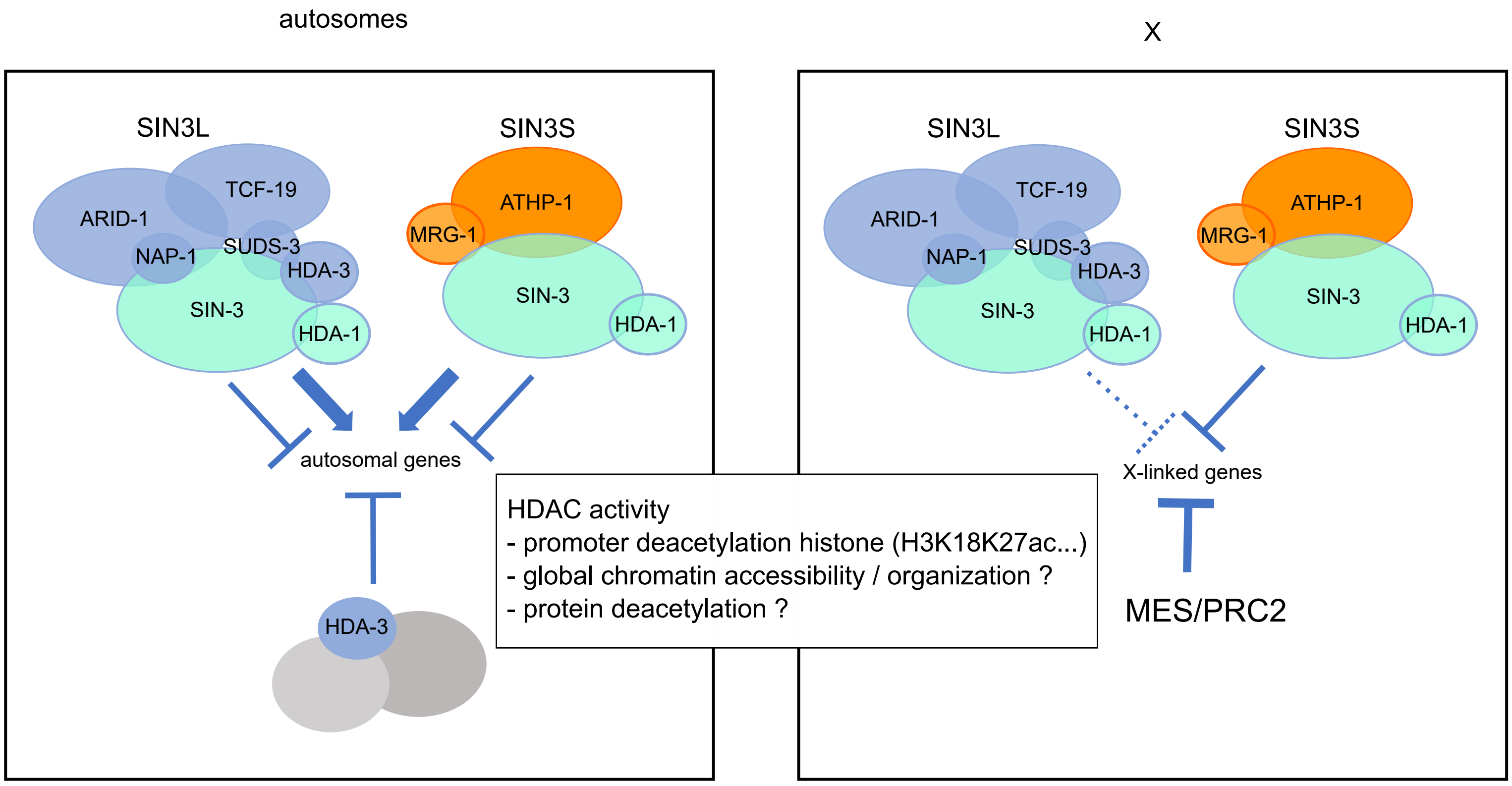

Fig. S9. Cartoon representation of SIN3S and SIN3L complexes and their effect of gene expression in the germline. On autosomal genes, SIN3L and SIN3S complexes activate or repress target genes, with a bias for gene activation. HDA-3, acting alone or in another complex, represses expression of additional genes. On the X chromosome, a SIN3S complex contributes to the silencing of X linked genes, either alone or in collaboration with the MES/PRC2 complex. SIN3L may also play a minor role in X gene silencing. HDAC activity associated with each complex may promote histone deacetylation at promoters, alter chromatin accessibility or chromatin organization, and target non-histone substrates. Arrows indicate activation, bars repression. The thickness of the lines indicates magnitude of the effect (expression of more or fewer genes altered in the absence of a given complex). The model does not imply any specific mechanism.

Table S1. Mass spectrometry-based characterization of SIN-3 and ARID-1 interactomes

Available for download at
https://journals.biologists.com/dev/article-lookup/doi/10.1242/dev.201755#supplementary-data

Table S2. List of significant misregulated genes in hda-3(ok1991) allele as compared to wild-type

Available for download at
https://journals.biologists.com/dev/article-lookup/doi/10.1242/dev.201755#supplementary-data

Table S3. Hypergeometric tests related to Figure 4B and to the repartition of sin-3(syb) upregulated oogenic genes on the X chromosome

Available for download at
https://journals.biologists.com/dev/article-lookup/doi/10.1242/dev.201755#supplementary-data

Table S4. List of primers used for genotyping

strain	primer name	sequence
syb2172	oLG53	AACAGCGTTGGAAATTTAAGGA
	oLG54	TTTCCAGCAAGGAACATGAA
	oLG55	GAAGCTGCTGGTGAATGTGA
syb2212	oLG61	TTGCAGTGTGGGTTTACGG
	oLG62	CCTTTCATTTCACAGGAGATGTT
tm4223	oFB59	GCGAGCCGAATACCTGACTC
	OFB62	CCTCGGCCATCAAGTACCTT
ok1991	CB389	tacttaaaggcgcacagacg
	hda-3 R1	AGCAGTTGTGCTCCAATGTG
	hda-3 R2	TCGAGACCCGTTGTTTGACG

Table S5. Sequences of smiFISH DNA probes

Available for download at
https://journals.biologists.com/dev/article-lookup/doi/10.1242/dev.201755#supplementary-data

Table S6. List of mean histone peptide abundances in *sin-3(syb2172)* and in wild-type (wt) young adults worms

Peptide	*sin-3(tm1276)*_mean	sin-3 _sd	wt_mean	wt_sd	Log2.FC	pval
H3K18acK23ac	0.0335575652499831	0.00180887408282901	0.0249882840710972	0.000410023599425965	0.425386209744721	0.00132242902646637
H3.3K27ac	0.00404100133919212	0.00069949266245853	0.00241528011331531	0.00028511356250372	0.742522313591324	0.0203364196956744
H3.3K27me1	0.00256638873132795	0.000168652757332963	0.00180823530924984	0.000342114754104861	0.505157281498187	0.0262274450758055
H4(K5K8K12K16)ac3	0.00859133719409992	0.00294431913002085	0.00420647627698246	0.000989044064919233	1.03027048832113	0.0708100369148078
H4(K5K8K12K16)ac2	0.0165558988426531	0.00365417849529556	0.0114446975735456	0.000273164435723216	0.532665998630554	0.0730843697403615
H3.3K27me1K36me1	0.0004788310275306	7.27598822176547e-05	0.000356673361281135	7.34283544134852e-05	0.424913169931642	0.110087314654162
H3K79me1	0.000336515331672776	0.000138917759729743	0.000600730787047685	1.53055729062035e-05	-0.836046367240991	0.116045584993093
H3K18me1K23me1	5.11438706526483e-05	8.73019471795978e-06	3.84735579631712e-05	7.44841146093102e-06	0.410694096694823	0.128400562486805
H3.3K36me1	0.00307345275598586	0.000235734243691193	0.00248678221624715	0.000489412906525282	0.305580141785629	0.134742543416522
H3(K18K23)ac	0.166677099096795	0.032174785467716	0.216951844038617	0.0390650087011052	-0.380318952537158	0.160436551493616
H4(K5K8K12K16)ac4	0.0104934709662109	0.00491616674131959	0.0053149166735416	0.00281534687806061	0.981372984014907	0.188532396502314
H3K27ac	0.00417313350162356	0.000570621721919821	0.00330406919898598	0.000896233889485998	0.336887171969448	0.229508315236609
H2B1K30me3	0.000672561157046867	0.000156409630404667	0.00180912643101981	0.00158541073805794	-1.4275558691176	0.284183473712587
H3K79me3	0.00536939940534031	0.00107653640469126	0.00376525880013259	0.00209865193030423	0.512011694390459	0.304101319166254
H4K20ac	0.000193452589772815	0.000102939011618523	0.000358215640912117	0.000225536212652673	-0.888848289226579	0.313817611031246
H3K27me1	0.0121617849453049	0.000646680392373322	0.0101480200663005	0.00315869623095562	0.261156706696155	0.340203047596258
H3K56ac	0.00087501189196222	0.000351009002260983	0.00127309543685194	0.000709446608819424	-0.540966044755604	0.432861165064425
H2A(K5K8K10K16)ac	0.0267714157432667	0.00348283575892293	0.0310609573896685	0.00800779784190069	-0.21440886397131	0.442803870688184
H2A(K5K8K10K16)ac3	0.00111785982185892	0.000412923870442918	0.00167394099928353	0.00138880147158144	-0.582509391224086	0.542570083313864
H3K18me1	0.0779406315858331	0.00710978276189134	0.0727683853701119	0.0122151918300352	0.0990638226683468	0.560615080314907
H3K27me2K36me1	0.132991241632724	0.0341198528190004	0.106135710521137	0.0707077578149003	0.325421089223879	0.585401330547285
H3K27me1K36me1	0.00260740882678224	7.41580896868481e-05	0.00242258370293277	0.000623572326060067	0.106070293732616	0.637042919779918
H4K20me1	0.0497622727650033	0.00839451460989925	0.0458876497816592	0.0120931267909154	0.116946459966435	0.67212978965677
H3K36me1	0.0133899807326497	0.00192533516804838	0.0125721721136975	0.00261644096467349	0.0909199567596994	0.685305571555693
H3K36me3	0.00928715736815369	0.00245615926124992	0.00791724779127956	0.00521559955114447	0.230238076106743	0.70174604750606
H2A(K5K8K10K16)ac2	0.00424045908943902	0.00133324773228585	0.00480007518297897	0.00210219669561111	-0.178836537455301	0.716840259120006
H3.3K27me2K36me1	0.0141537773070452	0.00760939720267201	0.0158002370694446	0.00491302727889955	-0.158759079517895	0.76861911240692
H3.3K36me3	0.00105969236626971	0.000602451865103167	0.00118368922863015	0.000390926746247393	-0.15964485435676	0.779796154570549
H3K79ac	0.0020969270190742	0.000303060396176319	0.00231720450246474	0.00200220439548655	-0.144108722257152	0.859728057703006
H2B1K30me1	0.00209542192617153	0.000305228323917585	0.00231532622977264	0.00200277746179323	-0.143974714981002	0.860022997541493
H3.3K27me3	0.0145776722882828	0.00142314324345944	0.0153946620971317	0.00952173286865288	-0.0786698279506223	0.915443880731859
H4(K5K8K12K16)ac	0.0438747938749314	0.0046059027668446	0.0444140152692456	0.00858843338690729	-0.0176226570927317	0.928261320197444
H3K27me2K36me2	6.92499851828365e-06	2.19477077551105e-06	7.09656561773089e-06	2.38599683929084e-06	-0.03530723890357	0.947152390738333
H3K23me1	0.00503462698259827	0.000558572026827529	0.00500003038437673	0.000742021285481406	0.00994803043620551	0.951652435444596
H3.3K27me2K36me2	2.08324215650613e-05	7.75682524403996e-06	2.18743251037883e-05	2.0303348098098e-05	-0.0704079573555815	0.952117505796686
H3K4me2	1.05598761856448e-05	6.11756180702776e-06	1.07733770178151e-05	3.5132255406554e-06	-0.0288776280770643	0.960708297221256
H3.3K27me2	0.000987409575675699	0.000470180576896098	0.00100001584047123	0.000583567079955403	-0.0183023115971963	0.978152078969147

Development • Supplementary information
