# Supplementary material for: SIN-3 acts in distinct complexes to regulate the germline transcriptional program in Caenorhabditis elegans
Source: Development. 2023 Oct 17;150(21):dev201755. doi: 10.1242/dev.201755 (PMC10617626; doi:10.1242/dev.201755)
Supplement: Supplementary file 1 [file develop-150-201755_review_history.pdf]

Development

SIN3 acts in distinct complexes to regulate the germline transcriptional program in *Caenorhabditis elegans*

Valerie J. Robert, Matthieu Caron, Loic Gely, Annie Adrait, Victoria Pakulska, Yohann Couté, Manon Chevalier, Christian G. Riedel, Cecile Bedet and Francesca Palladino
DOI: 10.1242/dev.201755

Editor: Swathi Arur

Review timeline

Original submission:	8 March 2023
Editorial decision:	4 April 2023
First revision received:	23 August 2023
Editorial decision:	7 September 2023
Second revision received:	13 September 2023
Editorial decision:	15 September 2023
Third revision received:	15 September 2023
Accepted:	18 September 2023

Original submission

First decision letter

MS ID#: DEVELOP/2023/201755

MS TITLE: SIN3 acts in distinct complexes to regulate the germline transcriptional program in C. elegans

AUTHORS: Matthieu Caron, Valerie J Robert, Loic Gely, Annie Adrait, Victoria Pakulska, Yohann Coute, Marion Chevalier, Christian G Riedel, Cecile Bedet, and Francesca Palladino

I have now received all the referees' reports on the above manuscript, and have reached a decision. The referees' comments are appended below, or you can access them online: please go to BenchPress and click on the 'Manuscripts with Decisions' queue in the Author Area.

As you will see, the referees express considerable interest in your work, but also provide recommendations to further improve the clarity and rigor of analysis. If you are able to revise the manuscript along the lines suggested, which may involve further experiments, I will be happy receive a revised version of the manuscript. Your revised paper will be re-reviewed by one or more of the original referees.

Please attend to all of the reviewers' comments and ensure that you clearly highlight all changes made in the revised manuscript. Please avoid using 'Tracked changes' in Word files as these are lost in PDF conversion. I should be grateful if you would also provide a point-by-point response detailing how you have dealt with the points raised by the reviewers in the 'Response to Reviewers' box. If you do not agree with any of their criticisms or suggestions please explain clearly why this is so.

Reviewer 1

Advance summary and potential significance to field

© 2023. Published by The Company of Biologists under the terms of the Creative Commons Attribution License (https://creativecommons.org/licenses/by/4.0/).

This interesting manuscript from Caron and colleagues investigates the importance of SIN-3 histone deacetylase complexes in C. elegans development focusing particularly on the germ line. The study addresses a conserved process regulating gene expression in development and will be of general interest to readers. Importantly, the authors recovered a deletion allele, syb2172, that allowed them to describe the sin-3 null phenotype. They demonstrate partial rescue by maternal expression in the M+Z- generation and complete sterility in the M-Z- generation. The authors isolate numerous protein interactors of SIN-3 using a mass spec approach, and they use these interactions to argue for two distinct SIN-3 complexes. They show that animals are sterile in the absence of pairs of interactors from the two proposed complexes, lending support to the idea of two distinct SIN-3 complexes. Their genetic and transcriptome data support the idea that the two complexes regulate at least somewhat distinct gene targets in the germ line. The authors also describe genetic interactions with components of the MES complex as well as smFISH and other experiments examining germline X chromosome gene expression.

Comments for the author

Points to address
Page 6: The authors should spell out more clearly their logic and assumptions behind assigning various interactors to different subunits. This is an important point in the paper and must be described more clearly. Any assumptions from earlier literature should be clearly spelled out.

Page 9, middle paragraph: It seems very likely that some differential gene expression is an indirect effect of the loss of SIN-3 activity, especially for genes where SIN-3 is not known to bind. This is obvious but should be stated anyway.

Page 11, line2, and Figure 4C: The authors do not include sin-3/sin-3 as a control for the sin-3/sin-3;mes-2/+ brood.

Figure 1A, D, F, G: Add labels to the images. Consistently indicate the distal tip with an "*" and label the various germline regions and features.

Page 13, 2nd paragraph: Sentence should read "Maternal effect sterility associated with sin-3 inactivation was revealed here using a CRISPR-Cas9 null allele, syb2172, …". It is vague to refer to syb2172 as loss-of-function.

Page 13, discussion of lin-15B expression at the bottom of the page: Although lin-15B mRNA is upregulated in both mes and sin-3 mutants, LIN-15B protein abundance may only be up-regulated in mes. This difference could explain why lin-15B loss-of-function suppresses the mes phenotype and not the sin-3 phenotype.

In the Introduction or Discussion, the authors should cite Checchi & Engebrecht (2011), who examined SIN-3 activity with respect to histone modifications and RNAPII association with unpaired chromosomes in the germ line. This earlier study has implications for the current work.

<u>Reviewer 2</u>

Advance summary and potential significance to field

SIN3 is a subunit of histone deacetylase complexes (HDAC) important for gene repression in eukaryotes. SIN3 functions in different forms of HDAC complexes. The authors addressed the roles of SIN3 within the different HDACs within a developmental context, using C. elegans. This is important because SIN3 and HDAC mutations are associated with several developmental diseases. The authors first used mass spec analysis to define the small and large HDACs that SIN-3 functions in. Loss of SIN-3 causes sterility, and each of SIN-3/HDAC complexes may affect germline development differently. The sterility in sin-3 mutants is caused by deregulation of the silenced X chromosome in a stochastic manner. Bulk-level changes in histone acetylation was observed in sin-3 mutant. Expression analysis in different mutants reveal how a single protein acts in distinct

© 2023. Published by The Company of Biologists under the terms of the Creative Commons Attribution License (https://creativecommons.org/licenses/by/4.0/).

complexes, and its loss cause heterogenous mis regulation of genes and result in distinct defects in germline transcriptional program.

Comments for the author

Major comments:
1-A figure like Figure 3F for the hda-3 misregulated genes, separated up&down, would be useful. While HDACs' direct function are assumed to be repression, there has been recent work suggesting that deacetylation may be needed for activation of transcription PMID: 31285436, thus some of the genes downregulated may be direct targets.
2-In sin-3 mutant the authors analyzed oogenic genes and X chromosome upregulation separately. Since X chromosomes have a distinct enrichment of genes with sex functions, it would be important to distinguishif the affect oogenic genes are disproportionately located on the X chromosome (that is, derepressed due to X upregulation) or located across the genome (that is, derepressed as part of the transcriptional program for oogenesis).

3-Performing motif analysis under SIN-3 binding peaks for genes up/down regulated in sin-3 mutants could add to this paper, by allowing the authors probe the mechanisms of HDAC S/L specificity. This could point to TFs that may be involved in recruiting SIN-3, as in Ume6 for recruiting Rpd3L complex in yeast. It could also help answer some of the redundant and nonredundant functions of the two HDAC complexes by contrasting motifs from sin-3 and hda-3 regulated genes.
4-Please clarify the interpretation of Figure 2 C? It suggests that S&L complexes have non redundant functions, rather than "Interestingly simultaneous inactivation of SIN3S and SIN3L complex subunits in both athp- 1;suds-3 and athp-1;hda-3 double mutant animals resulted in fully penetrant sterility (Figure 2C), suggesting redundant functions in the maintenance of germline function."
5-In addition, the authors should explain how the phenotypes of athp-1, hda-3, and suds-3 mutants and double mutants match that of sin-3? Do they show maternal effects similar to sin-3? Differences in the phenotypes of sin-3 and the double mutants may be due to the SIN-3 independent roles of each protein, thus important to document.

6-It would help the conclusion around "stochastic expression" if the authors can quantify the information in images in Figure 5B and provide more examples in the supplemental. To a non-expert, it is not clear how sharp the transition zone is in wild type versus mutant, and what the measure of "heterogeneity" is. In addition, for particularly in Figure 5B the mutant appears to show "patchy" expression in the late pachytene as well, which is not discussed. Is it possible that all expression appear more stochastic in the mutant because the germ cells at different meiotic stages are not as well positioned in the gonad?

7-The authors state in Figures 6B and S7B: the lack of H3K27Ac increase in sin-3 western blot may due to heterogeneity of the germ cells, since IF show increased H3K27Ac in sin-3 and SmiFISH is heterogenous. Therefore, the acetylation in western blots of hda-3 also may not reflect the true stage of acetylation in hda-3 worms. To conclude "No change in acetylation levels was observed in hda-3 mutant extracts, suggesting that another SIN-3 associated HDAC, most likely HDA-1, is responsible for deacetylation of these residues", the authors should perform experiments in hda-3, IF similar to Figure S7B and perhaps smiFISH, to analyze the heterogeneity of acetylation/gene expression in hda-3.

8-In table S3, please provide more information in the alleles. A supplemental file with the allele sequence across the junction of the deletion site would be critical to anyone who would like to use these CRISPR mutants.
9-Brood size scoring method is unclear. Does the final score shown in figures 1C, 2C, 4C, S6B include only embryos that are laid? Are hatching efficiencies differ in mutants?
10-Please clarify how the proteins found by IP-MS were assigned to different complexes. How were proteins assigned to SIN3L versus SIN3 S&L?
11-The manuscript would benefit from a "model" or "summary" figure explaining the main points in a cartoon form.
12-Data should be provided: Gene expression data are available at GEO with the accession XXXXX

Minor comments:

© 2023. Published by The Company of Biologists under the terms of the Creative Commons Attribution License (https://creativecommons.org/licenses/by/4.0/).

1-Please indicate which alleles are used on Figures A & B.
2-Note font difference Figure 3C
3-Figure 4A needs y axis description
4-Grammar: Loss of lin-15B in mes-4 mutants was shown to reduces X misexpression and prevent germline death (Cockrum and Strome, 2022).
5-Typo: Librairies were generated at the GenomEast Platform [IGBMC, Strasbourg, France] using the SMART-Seq v4
6-Missing syb2172 maintenance protocol in Material and Methods.
7-Consider different color scheme in Figure 3F.
8-Need scale bars for Figure 5.
9-Color of Figure 6A does not match descriptions.
10-Typo: refer to Figure S6A/S6B as Figure S5A/S5B in text.
11-Please provide citation for hda-3 does not show germline defects. (Page 10, at the end of “SIN-3 contributes to silencing of the silenced X chromosome in the germline” section)

Reviewer 3

Advance summary and potential significance to field

In figure 1, the authors show that sin3 null mutants have a maternal effect sterile phenotype. In figure 2 the authors perform mass spec IP of SIN3 and ARID1, to identify two different SIN3 complexes. They then use genetics in single and double mutants to show that the two complexes act distinctly and redundantly on fertility. In figure 3, the authors look at gene expression changes in the gonads of sin3 and hdac-3 mutants. They find that hdac-3 represses spermatogenesis genes, but largely does not overlap with sin3.
They also find that sin3 represses oogenesis genes. In figure 4, the authors show that sin3 misregulated genes overlap with x-linked genes, some of whom are also misregulated in mes-3 and mrg-1 mutants.
However, unlike in mes-4 mutants, sin3 mutant sterility is not suppressed by RNAi of lin-15B. They also find that sin3 genetically interacts with mes-2. In figure 4 they also find an increase in pol II on the x chromosome in sin3 mutants. There was no corresponding decrease in H3K27me3 on the x, although a few individual nuclei may have a decrease. Consistent with this potential derepression of x-linked genes, in figure 5 the authors find that lin-15B and nmy-1 are prematurely expressed in the transition zone, arguing that sin3 plays a role in preventing premature expression of genes on the x chromosome in the germline. In figure 6, the authors show an increase in histone acetylation by mass spec, western and staining in sin3 mutants. Overall, this paper provides a significant advance in our understanding of how repressive complexes function together with histone modifying enzymes to regulate the specification and timing of a developmental program (germline).

Comments for the author

This is a very thorough paper identifying two potentially distinct sin3 complexes and showing really consistent data supporting a functional interaction between sin3 and the function of the mes2,3,6 system in x-chromosome silencing shown by the Strome lab. Overall, I am very enthusiastic about this manuscript.
I think the finding of an interaction between sin3 and the mes2,3,6 system is particularly interesting. In light of this, perhaps the title and abstract should be slightly more focused on this?

Below are some additional comments that would be good to address.

The authors suggest that the fertility defect may be due to a combination of decrease in proliferation and increase in apoptosis. There certainly is a large decrease in number of germ cells, but many remain. So perhaps this is not necessarily consistent with the really small brood size. In addition, the increase in apoptosis is very small. Thus, it seems rather that the brood size decrease is more likely to be caused by germline disorganization, along with the failure to properly form oocytes and/or defects in fertilization of the oocytes that are formed. This is a particularly important distinction in light of the finding that oogenesis genes are upregulated in sin3 mutant gonads. Could this be the cause of the fertility defect.

© 2023. Published by The Company of Biologists under the terms of the Creative Commons Attribution License (https://creativecommons.org/licenses/by/4.0/).

The authors should make an attempt to examine by smFISH or another technique where the increase in oogenesis expression is. Is it just in oocytes? In proliferating germ cells? In sperm? Reciprocally, is there any evidence of sperm genes being expressed during oogenesis?

Acridine orange has been shown to stain residual bodies during spermatogenesis. In the disorganized germline, could the AO stained bodies be residual bodies? Are they next to sperm? This could indicate that spermatogenesis is still ongoing despite the switch to oogenesis. Showing the AO staining with corresponding DIC would resolve this issue.

The finding that the weaker allele tm1276 has many more transcriptional changes than syb2172 is perplexing. The authors suggest that this could be due to having maintained tm1276 over many generations. Does tm1276 have a germline mortality phenotype? The authors should at least state whether the brood size in figure 1 was carried out at the same generation as the RNAseq. If it was not, perhaps the authors should recount the brood size of tm1276 mutants that have been maintained as homozygotes for many generations, versus tm1276 mutants that have been balanced for several generations and then are counted in the first generation after being homozygous. This would give an indication of whether tm176 mutants have a germline mortality phenotype.

In figure 3D, it would be useful to calculate the fold change for the categories that are significantly enriched in observed over expected, so the magnitude of the change can be seen.

In figure 4B, the authors should calculate the statistical significance of the overlap.

Although mes-2 and mes-3 are related to mes-4, their functions are distinct. The authors should also examine whether sin3 has a genetic interaction with mes-4. Also, in figure 3B, the authors looked at the overlap with mes-3, while in figure 3C, they looked at the genetic interaction with mes-2. Was there a reason for this?

In figure 6B, the authors should calculate whether the observed changes are statistically significant.

In figure S7, the authors find that the increase in acetylation overall does not correlate with the increase in gene expression that they observe. This could be due to heterogeneity as the authors suggest. But it also could be due to the change occurring in either the gene body or in associated enhancers. This could be tested by performing ChIPseq. However, this is likely beyond the scope of this analysis, since it is not necessary to make the central conclusions of the paper. Nevertheless, the authors should probably add a sentence stating this alternative possibility.

In the discussion, the authors state that “On the silent X chromosome, we did not observe depletion of H3K27me3 in the absence of SIN3, arguing against a simple model in which desilencing results from loss of repressive chromatin.” While they did not observe a change in H3K27me3 by IF, there may still be a change that results in the effect. This might only be seen by H3K27me3 ChIPseq. Thus, while it is possible that the effect that they observe on the X is occurring solely through histone acetylation, it is also possible that SIN3 is affecting H3K27me3 either directly or indirectly and they do not observe it because of the resolution of IF. In light of this possibility, the authors should be careful not discount the model that SIN3 is functioning through H3K27me3.

Three typos

In the discussion maternal effect sterility is listed as maternal “effects”
In the section titled “SIN-3 inactivation results in precocious and stochastic transcription of autosomal and X-linked genes,” smFISH is listed as smiFISH
In the section titled “SIN-3 contributes to silencing of the silenced X chromosome in the germline,” the text says “to reduces” instead of “to reduce”

© 2023. Published by The Company of Biologists under the terms of the Creative Commons Attribution License (https://creativecommons.org/licenses/by/4.0/).

First revision

Author response to reviewers' comments

Reviewer 1 Advance Summary and Potential Significance to Field:
This interesting manuscript from Caron and colleagues investigates the importance of SIN-3 histone deacetylase complexes in C. elegans development focusing particularly on the germ line. The study addresses a conserved process regulating gene expression in development and will be of general interest to readers. Importantly, the authors recovered a deletion allele, syb2172, that allowed them to describe the sin-3 null phenotype. They demonstrate partial rescue by maternal expression in the M+Z- generation and complete sterility in the M-Z- generation. The authors isolate numerous protein interactors of SIN-3 using a mass spec approach, and they use these interactions to argue for two distinct SIN-3 complexes. They show that animals are sterile in the absence of pairs of interactors from the two proposed complexes, lending support to the idea of two distinct SIN-3 complexes. Their genetic and transcriptome data support the idea that the two complexes regulate at least somewhat distinct gene targets in the germ line. The authors also describe genetic interactions with components of the MES complex as well as smFISH and other experiments examining germline X chromosome gene expression.

Reviewer 1 Comments for the Author:
Points to address
Page 6: The authors should spell out more clearly their logic and assumptions behind assigning various interactors to different subunits. This is an important point in the paper and must be described more clearly. Any assumptions from earlier literature should be clearly spelled out.

We have now extensively rewritten the chapter "SIN-3 resides in distinct complexes", hopefully making it clearer how our results fit with published data. We also added a Venn diagram (Figure S2C) showing the overlap between SIN-3, ARID-1, and our previously published CFP-1 co-IP/mass spec experiments that should help understand our logic in assigning subunits to SIN3L or S complexes.

Page 9, middle paragraph: It seems very likely that some differential gene expression is an indirect effect of the loss of SIN-3 activity, especially for genes where SIN-3 is not known to bind. This is obvious but should be stated anyway.

We completely agree. On p. 9 we have added the phrase "Our expression analysis does not allow us to distinguish direct from indirect targets of SIN-3, and it is likely that many of identified changes in gene expression are an indirect result of SIN-3 loss".

Page 11, line2, and Figure 4C: The authors do not include sin-3/sin-3 as a control for the sin-3/sin-3;mes-2/+ brood.

This figure has now been completely changed and includes the sin-3/sin-3 control. Please see response to reviewer 3 below.

Figure 1A, D, F, G: Add labels to the images. Consistently indicate the distal tip with an "*" and label the various germline regions and features.

This was done

Page 13, 2nd paragraph: Sentence should read "Maternal effect sterility associated with sin-3 inactivation was revealed here using a CRISPR-Cas9 null allele, syb2172, …". It is vague to refer to syb2172 as loss-of-function.

This was done

© 2023. Published by The Company of Biologists under the terms of the Creative Commons Attribution License (https://creativecommons.org/licenses/by/4.0/).

Page 13, discussion of lin-15B expression at the bottom of the page: Although lin-15B mRNA is upregulated in both mes and sin-3 mutants, LIN-15B protein abundance may only be up-regulated in mes. This difference could explain why lin-15B loss-of-function suppresses the mes phenotype and not the sin-3 phenotype.

Thank you to the reviewer for pointing this out. On page 14 we have added the sentence "Alternatively, LIN-15B protein abundance may only increase in mes, but not sin-3 or hda-3 mutants".

In the Introduction or Discussion, the authors should cite Checchi & Engebrecht (2011), who examined SIN-3 activity with respect to histone modifications and RNAPII association with unpaired chromosomes in the germ line. This earlier study has implications for the current work.

Thank you for the reminder. On p.14 we added this and another reference that should have been included
"Loss of SIN-3 may also influence germline gene expression and overall chromatin organization, as suggested by altered distribution of repressive H3K9me2 in sin-3(tm1276) mutant animals (Checchi and Engebrecht, 2011; She et al., 2009)".

*****Reviewer 2 Advance Summary and Potential Significance to Field:

SIN3 is a subunit of histone deacetylase complexes (HDAC) important for gene repression in eukaryotes. SIN3 functions in different forms of HDAC complexes. The authors addressed the roles of SIN3 within the different HDACs within a developmental context, using C. elegans. This is important because SIN3 and HDAC mutations are associated with several developmental diseases. The authors first used mass spec analysis to define the small and large HDACs that SIN-3 functions in. Loss of SIN-3 causes sterility, and each of SIN-3/HDAC complexes may affect germline development differently. The sterility in sin-3 mutants is caused by deregulation of the silenced X chromosome in a stochastic manner. Bulk-level changes in histone acetylation was observed in sin-3 mutant. Expression analysis in different mutants reveal how a single protein acts in distinct complexes, and its loss cause heterogenous mis regulation of genes and result in distinct defects in germline transcriptional program.

Reviewer 2 Comments for the Author:

Major comments:

1-A figure like Figure 3F for the hda-3 misregulated genes, separated up&down, would be useful. While HDACs' direct function are assumed to be repression, there has been recent work suggesting that deacetylation may be needed for activation of transcription PMID: 31285436, thus some of the genes downregulated may be direct targets.

We thank the review for pointing out this article, which we now cite. Other articles pointing to a role for SIN3 and HDACS in gene activation were cited elsewhere in our previous version (p15) "Our expression profiling shows that, as in other systems, loss of SIN-3 results in both repression and activation of gene expression (Saha et al 2016; Saunders et al 1017; van Oevelen et al., 2010), consistent with the presence of HDACs at promoters of actively transcribed as well as repressed genes (Wang et al., 2009)".

Because HDA-3 ChIP-seq data is not available to look at how hda-3 up and down genes correlate with HDA-3 binding, we instead looked at SIN-3 binding on these genes (Figure 3E). This analysis shows that while the majority of hda-3 up genes are not bound by SIN-3, consistent with hda-3 activity independent of SIN-3, more than 50% of hda-3 down genes are in fact associated with SIN-3 binding.
We modified the text as follows
"Our expression analysis does not allow us to distinguish direct from indirect targets of SIN-3, and it is likely that many of the identified changes in gene expression are an indirect result of SIN-3 loss. To identify potential direct targets of SIN3 regulation, we compared our list of misregulated genes in sin-3(syb2172) mutants to a published list of SIN-3 binding sites obtained by ChIP-seq (Beurton et al., 2019). When considering all misexpressed genes, SIN-3 binding is observed on the

© 2023. Published by The Company of Biologists under the terms of the Creative Commons Attribution License (https://creativecommons.org/licenses/by/4.0/).

promoter of both up- and down-regulated genes, with a bias towards downregulated genes (Figure 3E). Similar analysis on hda-3 misregulated genes revealed that a majority of hda-3 downregulated genes are bound by SIN-3 (290/489). Of these, a fraction are commonly downregulated in sin-3 (167/290): these likely represent targets of a SIN-3/HDA-3 complex, consistent with a role for deacetylation in gene activation (Gryder et al., 2019). As expected, the large majority of genes upregulated in hda-3 are not associated with SIN-3 binding, in agreement with their regulation being independent of sin-3 (Figure 3B).

2-In *sin-3* mutant the authors analyzed oogenic genes and X chromosome upregulation separately. Since X chromosomes have a distinct enrichment of genes with sex functions, it would be important to distinguish if the affect oogenic genes are disproportionately located on the X chromosome (that is, derepressed due to X upregulation) or located across the genome (that is, derepressed as part of the transcriptional program for oogenesis).

Thank you for pointing this out. We have done this analysis and added it in a supplementary table (Table S3). Hypergeometric test reveals that X-linked oogenic genes are not preferentially upregulated in sin-3 mutants with respect to other gene classes: the same expected bias is observed as in wild-type (hypergeometric test, Table S3. We comment on this in the text as follows:

"Because oogenic genes are enriched on the X (Ortiz et al 2014), we asked whether their overrepresentation in the set of sin-3 upregulated genes reflects the upregulation of the X, or a general deregulation of the oogenic program. Although the expected bias for oogenic genes was observed, this was no larger than expected (Table S3), and upregulated genes were not uniquely oogenic. This suggest that derepression of the X, rather than a general deregulation of the oogenic program per se, most likely accounts for the enrichment of this class of genes in the set of sin-3 upregulated genes".

3-Performing motif analysis under SIN-3 binding peaks for genes up/down regulated in sin-3 mutants could add to this paper, by allowing the authors probe the mechanisms of HDAC S/L specificity. This could point to TFs that may be involved in recruiting SIN-3, as in Ume6 for recruiting Rpd3L complex in yeast. It could also help answer some of the redundant and nonredundant functions of the two HDAC complexes by contrasting motifs from sin-3 and hda-3 regulated genes.

Motif analysis using the MEME-ChIP algorithm (https://meme-suite.org/meme/tools/meme-chip) on all genes bound by SIN-3 identified a T-rich motif (TTTTYWTYRWTTTTY) in almost half of SIN-3 binding sites on promoter regions, regardless of the transcriptional status of the underlying gene (up, down or not misregulated). This motif is similar to the binding site of the CEH-38 transcription factor, but no additional information is available of the specificity of this factor or its interactors, and CEH-38 was not found in our list of SIN-3 interactors. ARID-1 or C01G6.5 are two transcriptional factors isolated in our co-IP experiments that could potentially recruit SIN-3 to chromatin, but ChIP-seq data is not available for these. Because this information does not provide any significant insight on the specificity of SIN-3 binding and function, we chose not to mention it in the text. In future experiments we plan to carry out ChIP-seq on tagged ARID-1, SUDS-3 and ATHP-1 in order to identify targets of the SIN-3L and SIN-3S complexes.

4-Please clarify the interpretation of Figure 2 C? It suggests that S&L complexes have non redundant functions, rather than "Interestingly simultaneous inactivation of SIN3S and SIN3L complex subunits in both athp- 1;suds-3 and athp-1;hda-3 double mutant animals resulted in fully penetrant sterility (Figure 2C), suggesting redundant functions in the maintenance of germline function."

sin-3 null mutants are fully sterile, while single mutants of SIN3 complex subunits (S or L) are either fully fertile (suds-3) or have only slightly reduced fertility (athp-1 and hda-3). However, when both L and S complex subunits are inactivated (ie athp-1;suds-3 and athp-1; hda-3), animals are completely sterile. This synergism suggests that both L and S complex subunits contribute to fertility, and both complexes have to be inactivated to reproduce the sterility observed in single sin-3 mutants, suggesting functional redundance. However, the actual mechanisms may be quite distinct, so the reviewer is correct that redundancy may not be the right term to use. We therefore changed our phrasing as follows:

© 2023. Published by The Company of Biologists under the terms of the Creative Commons Attribution License (https://creativecommons.org/licenses/by/4.0/).

“Interestingly simultaneous inactivation of SIN3S and SIN3L complex subunits in both athp-1;suds-3 and athp-1;hda-3 double mutant animals resulted in fully penetrant sterility (Figure 2C). This genetic interaction suggests common roles in the maintenance of germline function”.

5-In addition, the authors should explain how the phenotypes of athp-1, hda-3, and suds-3 mutants and double mutants match that of sin-3? Do they show maternal effects similar to sin-3? Differences in the phenotypes of sin-3 and the double mutants may be due to the SIN-3 independent roles of each protein, thus important to document.

We did indeed try to analyze these germlines. However, we were unable to maintain the double mutants because of their sterility, and constructing doubly balanced strain constitutes a significant amount of work that will not necessarily provide clear answers to the question. As we discuss, it is highly likely that athp-1, suds-3 and had-3 have additional functions independent of sin-3, since double mutants are completely sterile, while the single sin-3 mutant manages to lay the few occasional eggs. The few double mutant germline that we were able to look at were highly disorganized and it was difficult to discern any germline features.

6-It would help the conclusion around “stochastic expression” if the authors can quantify the information in images in Figure 5B and provide more examples in the supplemental. To a non-expert, it is not clear how sharp the transition zone is in wild type versus mutant, and what the measure of “heterogeneity” is. In addition, for particularly in Figure 5B the mutant appears to show “patchy” expression in the late pachytene as well, which is not discussed.

We have made substantial changes to this figure and added other examples. To facilitate the interpretation of the images, we increased their size in the new figure 5. We also provided additional examples of what we define as “stochastic expression” in Figure S7.
While we agree with the reviewer that quantifying smFISH spots would provide useful additional information, this presents several challenges. In fact closer examination of the images that we now provide shows that the increase in smFISH spots in sin-3 mutant germlines is mostly observed in the cytoplasm surrounding individual nuclei. Individual cytoplasmic spots could be counted on a per cell basis, but this would require automation using custom MATLAB codes (as described in Lee et al. eLife 2016;5:e18370. DOI: 10.7554/eLife.18370). This will be the subject of a future study beyond the scope of this paper, using both intronic and exonic probes.

As an approximation, we instead applied a semi-quantitative approach, visually counting lin-15B smiFISH spots on representative images of both wild-type and sin-3(syb2172) germlines. 57% of wild-type pachytene nuclei (n=158) had spots, and this increased to 70% for sin-3(syb2172) pachytene nuclei (n=73)(hypergeometric p-value=0.002). This rough estimate shows that more transcripts are indeed detected in sin-3 mutants than wildtype. However, as this is a very approximative estimate, we prefer not to include it in the manuscript.

The “patchy”expression that we refer to as stochastic expression is observed for both lin-15B and nmy-1, and we now show that it is observed in both early (we no longer refer to transition zone nuclei because the DAPI morphology is more consistent with early pachytene) and late pachytene, and contrasts with the nearly uniform field of mRNAs observed in wildtype. For both mRNAs, a group of nuclei with very few or no smFISH spots is found just adjacent to, or surrounded by areas with many cytoplasmic spots. Because free diffusion of mRNAs between cells through the rachis (the central cytoplasmic core of the germline) is not observed (doi: 10.7554/eLife.18370), these observations further strengthen the argument that not all nuclei produce more transcripts, and is consistent with a stochastic effect on transcription.

Is it possible that all expression appear more stochastic in the mutant because the germ cells at different meiotic stages are not as well positioned in the gonad?

We considered this possibility. However, if this were the case the DNA of smFISH negative cells immediately adjacent to cells with many spots should show features of another state by DAPU staining. This is not the case: in the images of sin-3 mutants in Figure 5, in each panel we added arrows to show two examples of nuclei in the “bowl of spaghetti” pachytene stage, one of them with many spots and another one with few or none.

© 2023. Published by The Company of Biologists under the terms of the Creative Commons Attribution License (https://creativecommons.org/licenses/by/4.0/).

7-The authors state in Figures 6B and S7B: the lack of H3K27Ac increase in sin-3 western blot may due to heterogeneity of the germ cells, since IF show increased H3K27Ac in sin-3 and SmiFISH is heterogenous. Therefore, the acetylation in western blots of hda-3 also may not reflect the true stage of acetylation in hda-3 worms. To conclude “No change in acetylation levels was observed in hda-3 mutant extracts, suggesting that another SIN-3 associated HDAC, most likely HDA-1, is responsible for deacetylation of these residues”, the authors should perform experiments in hda-3, IF similar to Figure S7B and perhaps smiFISH, to analyze the heterogeneity of acetylation/gene expression in hda-3.

We chose to directly address the question of whether HDAC-1 is responsible for the increase in H3K18Ac associated with loss of sin-3 by performing Western blot analysis on animals that allow the auxin-inducible degradation of hda-1 (hda-1 null mutants are not viable). These blots (new Figure S8A) show a clear increase in H3K18Ac (and to a lesser extent H3K27Ac) following hda-1 auxin degradation, and confirm the absence of an effect in hda-3 mutant animals, supporting a prominent role for HDA-1, but not HDA-3, in the observed increase observed in sin-3(tm1276) animals by sequencing and WB. These new blots further show that H3K18Ac is also increase in syb2172 null mutants, further supporting the involvement of SIN-3 in deacetylation of this mark. We note that although results are variable between experiments, the trend between individual biological replicas is the same. Although we cannot rule out a minor role for hda-3 not detectable by WB analysis, we feel that our western blot analysis support the hypothesis that hda-1 is the major contributor to the sin-3 dependent HDAC activity we detect. In future we plan to perform HDAC-3 histone mass spec experiments to identify residues targeted by this HDAC.

We did not carry out smFISH experiments of hda-3 mutants to address the question of heterogeneity as these experiments require substantial time investment and are part of a future study. Also because the student who carried out the IF experiments left the lab, we were unable to perform these experiments within the allotted time.

8-In table S3, please provide more information in the alleles. A supplemental file with the allele sequence across the junction of the deletion site would be critical to anyone who would like to use these CRISPR mutants. SIN-3 and SUDS-3

We have added a file on github (https://gitbio.ens-lyon.fr/cbedet/supplemental_files_cbedet/) which contains the sequence of suds-3 and sin-3 alleles and the junction of the respective deletion.

9-Brood size scoring method is unclear. Does the final score shown in figures 1C, 2C, 4C, S6B include only embryos that are laid? Are hatching efficiencies differ in mutants?

We scored the total number of progeny produced. Hatching efficiency was not affected in the different mutant context (no embryonic lethality).

10-Please clarify how the proteins found by IP-MS were assigned to different complexes. How were proteins assigned to SIN3L versus SIN3 S&L?

This part was rewritten to take into accounts comments by this reviewer and reviewer 1. We also added a venn diagram to show overlap between the proteins identified in the SIN-3 and ARID-1 IP-MS experiment performed here, and the CFP-1 IP-MS data from our previous work (Beurton et al 2019) (Figure S2C), since this information helped us establish the composition of the different complexes.

11-The manuscript would benefit from a “model” or “summary” figure explaining the main points in a cartoon form.

We have added a cartoon to summarize the data presented in supplementary Figure S9.

12-Data should be provided: Gene expression data are available at GEO with the accession XXXXX

This has been done and the GEO access is provided: GSE227499

© 2023. Published by The Company of Biologists under the terms of the Creative Commons Attribution License (https://creativecommons.org/licenses/by/4.0/).

Minor comments:

1-Please indicate which alleles are used on Figures A & B.
We indicated allele used in figure legends everywhere

2-Note font difference Figure 3C
This was done

3-Figure 4A needs y axis description
This was done

4-Grammar: Loss of lin-15B in mes-4 mutants was shown to reduces X misexpression and prevent germline death (Cockrum and Strome, 2022).
Done

5-Typo: Librairies were generated at the GenomEast Platform [IGBMC, Strasbourg, France] using the SMART-Seq v4
Done

6-Missing syb2172 maintenance protocol in Material and Methods.
This was added to the Material and Methods section

7-Consider different color scheme in Figure 3F.
This was done

8-Need scale bars for Figure 5.
This was done

9-Color of Figure 6A does not match descriptions.
Done

10-Typo: refer to Figure S6A/S6B as Figure S5A/S5B in text.
Done

11-Please provide citation for hda-3 does not show germline defects. (Page 10, at the end of "SIN-3 contributes to silencing of the silenced X chromosome in the germline" section)
This is shown in figure S3. We have added the information:

"We found that lin-15B(RNAi) had no effect on the fertility of sin-3 mutants (Figure S6B), while its upregulation in hda-3 mutants is not associated with any obvious germline defects (Figure S3), suggesting that additional mechanisms contribute to sterility in the absence of sin-3".

Reviewer 3 Advance Summary and Potential Significance to Field:

In figure 1, the authors show that sin3 null mutants have a maternal effect sterile phenotype. In figure 2, the authors perform mass spec IP of SIN3 and ARID1, to identify two different SIN3 complexes. They then use genetics in single and double mutants to show that the two complexes act distinctly and redundantly on fertility. In figure 3, the authors look at gene expression changes in the gonads of sin3 and hdac-3 mutants. They find that hdac-3 represses spermatogenesis genes, but largely does not overlap with sin3. They also find that sin3 represses oogenesis genes. In figure 4, the authors show that sin3 misregulated genes overlap with x-linked genes, some of whom are also misregulated in mes-3 and mrg-1 mutants. However, unlike in mes-4 mutants, sin3 mutant sterility is not suppressed by RNAi of lin-15B. They also find that sin3 genetically interacts with mes-2. In figure 4 they also find an increase in pol II on the x chromosome in sin3 mutants. There was no corresponding decrease in H3K27me3 on the x, although a few individual nuclei may have a decrease. Consistent with this potential derepression of x-linked genes, in figure 5 the authors find that lin-15B and nmy-1 are prematurely expressed in the transition zone, arguing that sin3 plays a role in preventing premature expression of genes on the x chromosome in the germline. In figure 6,

© 2023. Published by The Company of Biologists under the terms of the Creative Commons Attribution License (https://creativecommons.org/licenses/by/4.0/).

the authors show an increase in histone acetylation by mass spec, western and staining in sin3 mutants. Overall, this paper provides a significant advance in our understanding of how repressive complexes function together with histone modifying enzymes to regulate the specification and timing of a developmental program (germline).

Reviewer 3 Comments for the Author:

This is a very thorough paper identifying two potentially distinct sin3 complexes and showing really consistent data supporting a functional interaction between sin3 and the function of the mes2,3,6 system in x-chromosome silencing shown by the Strome lab. Overall, I am very enthusiastic about this manuscript. I think the finding of an interaction between sin3 and the mes2,3,6 system is particularly interesting. In light of this, perhaps the title and abstract should be slightly more focused on this?

While we agree that this is a very interesting aspect of our work, we feel that the present data does not go far enough to establish how SIN-3 and PRC2 are interacting, and prefer not to overinterpret our data. However, we modified the abstract as follows:

"Single cell smFISH reveals that in sin-3 mutants, the X chromosome becomes re-expressed prematurely and in a stochastic manner in individual germ cells, suggesting a role for SIN-3 in its silencing."

Below are some additional comments that would be good to address.

The authors suggest that the fertility defect may be due to a combination of decrease in proliferation and increase in apoptosis. There certainly is a large decrease in number of germ cells, but many remain. So perhaps this is not necessarily consistent with the really small brood size. In addition, the increase in apoptosis is very small. Thus, it seems rather that the brood size decrease is more likely to be caused by germline disorganization, along with the failure to properly form oocytes and/or defects in fertilization of the oocytes that are formed. This is a particularly important distinction in light of the finding that oogenesis genes are upregulated in sin3 mutant gonads. Could this be the cause of the fertility defect. The authors should make an attempt to examine by smFISH or another technique where the increase in oogenesis expression is. Is it just in oocytes? In proliferating germ cells? In sperm? Reciprocally, is there any evidence of sperm genes being expressed during oogenesis?

We agree with the reviewer that additional, more general defects in the germline transcriptional program may contribute to the sterility of sin-3 mutants. To ask where the increase in oogenesis expression is, we performed smFISH analysis on the oogenic gene vrsa-1. While in wild-type we observed a signal starting in pachytene, but never in the distal region, as expected, in sin-3 mutant germlines signal was detected in the distal germline, consistent with precocious expression of oogenic genes (Figure S5B). We did not have the spermatheca in the dissected germlines, nor did we perform smiFISH with sperm genes, so in the present work we cannot directly answer this question. However, spermatogenic genes were not overrepresented in our set of upregulated genes in sin-3 mutants (Figure 2D).
Arguing against overall defects in germline organization, immunostaining did not reveal proximal mitotic figures, and HIM-3 staining was similar to wild-type (Figure 1F). However, the "Gogo" phenotype observed in some animals is consistent with a defect in the developmental switch regulating the transition from late pachytene to oogenesis is, as mentioned at the end of the section "Reduced proliferation of progenitor cells in sin-3 mutant germlines". Taking all this into account, we have accordingly modified the text:

"Altogether, these results suggest that a decrease in germ cell proliferation, in combination with a small increase in apoptosis, contribute to the sterility of sin-3 mutants. In addition, more subtle defects in germline organization may also contribute to this loss of fertility, as revealed by the "Gogo" phenotype observed in some animals".

Acridine orange has been shown to stain residual bodies during spermatogenesis. In the disorganized germline, could the AO stained bodies be residual bodies? Are they next to sperm? This

© 2023. Published by The Company of Biologists under the terms of the Creative Commons Attribution License (https://creativecommons.org/licenses/by/4.0/).

could indicate that spermatogenesis is still ongoing despite the switch to oogenesis. Showing the AO staining with corresponding DIC would resolve this issue.

Unfortunately we do not have the corresponding DIC staining. However, the AO stained bodies we observe are always in the distal or bend region. We never observed any staining in the proximal region. An overexposed image (added in supplementary Figure S1) of the gonads shown in Figure 1G shows no signal in the proximal gonad, where oocytes are apparent. It is therefore highly unlikely that they AO staining corresponds to residual bodies

The finding that the weaker allele tm1276 has many more transcriptional changes than syb2172 is perplexing. The authors suggest that this could be due to having maintained tm1276 over many generations. Does tm1276 have a germline mortality phenotype? The authors should at least state whether the brood size in figure 1 was carried out at the same generation as the RNAseq. If it was not, perhaps the authors should recount the brood size of tm1276 mutants that have been maintained as homozygotes for many generations, versus tm1276 mutants that have been balanced for several generations and then are counted in the first generation after being homozygous. This would give an indication of whether tm176 mutants have a germline mortality phenotype.

We have thought about this question and agree that the results are rather surprising. However, several variables need to be taken into account when comparing the two data sets: 1/ Protocols used for mRNA extraction were different : extraction was from approximately 100 gonads for tm1276, using the protocol given by the PicoPure kit (Life Technology, # 12204-01), but only 10-12 gonads for syb using Smart-seq 2 protocol; 2/ Librairies construction was different for each set : mRNA selection was polyA for syb, ribozero for tm1276 ; and importantly 3/ the depth of reads was not equal in both experiments. Because the coverage was higher for the tm1276 dataset (28,2M) than for the syb2172 (23,5M), we are likely to have missed many genes with a lower base mean in the latter dataset. Taking into account all these elements, we believe this to be a more likely explanation than the maintenance of the two strains. Furthermore, tm1276 mutants do not show a Mrt phenotype, as we have shown previously (Beurton et al. 2019) and in this work. We have modified this part of the text accordingly:

"The difference in the total number of misregulated genes may be attributed to the specific allele used, or to differences in sample preparation and processing. All subsequent analyses were carried out on the list of genes misregulated in the sin-3(syb2172) null allele".

In figure 3D, it would be useful to calculate the fold change for the categories that are significantly enriched in observed over expected, so the magnitude of the change can be seen.
We have done this and added the information on the graph

In figure 4B, the authors should calculate the statistical significance of the overlap.
We added a supplementary Table S3 with hypogeometric test results for all overlaps.

Although mes-2 and mes-3 are related to mes-4, their functions are distinct. The authors should also examine whether sin3 has a genetic interaction with mes-4. Also, in figure 3B, the authors looked at the overlap with mes-3, while in figure 3C, they looked at the genetic interaction with mes-2. Was there a reason for this?

We repeated these genetic analyses, using both mes-2 and mes-4 mutant alleles. Our interpretation of the results varies slightly from the previous one. In particular, we were unable to reproduce the sterility of sin-3/+; mes-2/mes-2 animals reported in the previous version: we are now confident that these animals are instead fertile. We believe this discrepancy resulted from errors in PCR genotyping sin-3, and that the animals scored as sterile were in fact sin-3/sin-3 M-Z-. sin-3/*sin-3*; *mes-2/mes-2 animals that inherited maternal SIN-3 and MES-2 proteins were not recovered, as in our previous experiments, suggesting a genetic interaction leading to unviable zygotes. As suggested by the reviewer, we also compared the fertility of +/+; mes-4/mes-4, sin-3/+; mes-4/mes-4 and sin-3/sin-3; mes-4/mes-4 mutants. We found that while +/+; mes-4/mes-4 and sin-3/+; mes-4/mes-4 mutants were fertile, sin-3/sin-3; mes-4/mes-4 animals inheriting both maternal SIN-3 and MES-4 protein were sterile. This genetic interaction is consistent with our previous conclusion that SIN-3 and MES act in parallel pathways to promote fertility. We*

© 2023. Published by The Company of Biologists under the terms of the Creative Commons Attribution License (https://creativecommons.org/licenses/by/4.0/).

accordingly also altered the venn diagram showing overlap of sin-3, mes-3 and mrg-1 targets on the X (mes-2 data is not available).

In figure 6B, the authors should calculate whether the observed changes are statistically significant.

We have performed a non parametric kruskal-wallis test which shows that the observed changes can't be considered as statistically significant (p values for k9ac = 0.1478193/ for k27ac = 0.09508908 / for K18ac= 0.1712371). However, in sin-3 mutants, the same slight increase in H3K18Ac and H3K27Ac but not in H3K9Ac was observed in both western blots, supporting the mass spec results. We have modified the manuscript to moderate this conclusion:
"Semi-quantitative western-blot analysis using antibodies that detect acetylation marks on both histone H3 and H3.3 showed a slight increased acetylation on K27 and K18, but not K9 in total extracts from sin-3(tm1276) mutant young adults (Figure 6B)."

In figure S7, the authors find that the increase in acetylation overall does not correlate with the increase in gene expression that they observe. This could be due to heterogeneity as the authors suggest. But it also could be due to the change occurring in either the gene body or in associated enhancers. This could be tested by performing ChIPseq. However, this is likely beyond the scope of this analysis, since it is not necessary to make the central conclusions of the paper. Nevertheless, the authors should probably add a sentence stating this alternative possibility.

We added the sentence
"Alternatively, changes in these marks may occur elsewhere in the genome and their detection would require genome-wide approaches".

In the discussion, the authors state that "On the silent X chromosome, we did not observe depletion of H3K27me3 in the absence of SIN3, arguing against a simple model in which desilencing results from loss of repressive chromatin." While they did not observe a change in H3K27me3 by IF, there may still be a change that results in the effect. This might only be seen by H3K27me3 ChIPseq. Thus, while it is possible that the effect that they observe on the X is occurring solely through histone acetylation, it is also possible that SIN3 is affecting H3K27me3 either directly or indirectly and they do not observe it because of the resolution of IF. In light of this possibility, the authors should be careful not discount the model that SIN3 is functioning through H3K27me3.

We thank the reviewer for pointing this out and we have nuanced this part of the discussion
"On the silent X chromosome, we did not detect depletion of H3K27me3 in the absence of SIN-3, arguing against a simple model in which desilencing results from loss of repressive chromatin. Nonetheless, loss of SIN-3 may result in alterations in the H3K27me3 landscape that are too small to be detected by immunofluorescence"

Three typos

In the discussion maternal effect sterility is listed as maternal "effects"

In the section titled "SIN-3 inactivation results in precocious and stochastic transcription of autosomal and X-linked genes," smFISH is listed as smiFISH

In the section titled "SIN-3 contributes to silencing of the silenced X chromosome in the germline," the text says "to reduces" instead of "to reduce"

These were corrected

Second decision letter

MS ID#: DEVELOP/2023/201755

© 2023. Published by The Company of Biologists under the terms of the Creative Commons Attribution License (https://creativecommons.org/licenses/by/4.0/).

MS TITLE: SIN3 acts in distinct complexes to regulate the germline transcriptional program in C. elegans

AUTHORS: Valerie J Robert, Matthieu Caron, Loic Gely, Annie Adrait, Victoria Pakulska, Yohann Coute, Marion Chevalier, Christian G Riedel, Cecile Bedet, and Francesca Palladino

I have now received all the referees reports on the above manuscript, and have reached a decision. The referees' comments are appended below, or you can access them online: please go to BenchPress and click on the 'Manuscripts with Decisions' queue in the Author Area.

The overall evaluation is positive and we would like to publish a revised manuscript in Development, however, there are some textual edits that the reviewers suggest, which I think will improve the readability of the manuscript and invite you to incorporate them into the manuscript. I do not expect to send the manuscript back to the reviewers, but I will look at it myself. So, Please attend to all of the reviewers' comments in your revised manuscript and detail them in your point-by-point response. If you do not agree with any of their criticisms or suggestions explain clearly why this is so. If it would be helpful, you are welcome to contact us to discuss your revision in greater detail. Please send us a point-by-point response indicating your plans for addressing the referees' comments, and we will look over this and provide further guidance.

Reviewer 1

Advance summary and potential significance to field

As indicated in my review of the initial submission, this manuscript investigates the importance of SIN-3 histone deacetylase complexes in C. elegans development focusing particularly on the germ line. The study addresses a conserved process regulating gene expression in development and will be of general interest to readers. The authors provide evidence for two distinct SIN-3 complexes in C. elegans and identify sets of mRNAs whose expression levels are altered in the absence of SIN-3 activity.

Comments for the author

Resubmitted ms from Robert, Caron et al., "SIN3 acts in distinct complexes to regulate the germline transcriptional program in C. elegans"

In this resubmitted manuscript, the authors have addressed many of the comments from the three reviewers regarding the initial submission. The manuscript would benefit from the authors addressing a few more points.

Comments on the current version

1) Fig. S3: The authors state on page 7 that mutations in athp-1, hda-3, and suds-3 have "no obvious defects" to account for reduced brood size, and they provide images in Fig. S3 as evidence. However, these germlines in fact do not look normal. The athp-1 germline has tightly stacked oocytes and a few apparently unfertilized oocytes in the uterus; either this animal is depleted of sperm (early) or did not make sperm. The hda-3 germline has a set of small, atypical cells located at about the spermatheca; it is unclear if these are abnormal spermatocytes and/or abnormal oocytes. This phenotype resembles the "gogo" phenotype shown in Fig. S1B. The suds-3 germline appears very small, although the distal end may be obscured by the intestine.
2) Page 4, top several lines: The authors state that tm1276 is a deletion of exon 3. It is important that the authors also tell us if this an in-frame deletion. If so, then this might help to explain why tm1276 has a greater impact on the transcriptome than does syb2172 (discussed on page 8). For example, the deleted protein may have residual and/or unregulated activity that alters gene expression.

In addition, the description of syb2172 strain maintenance is confusing. The authors state that "…syb2172 homozygous animals were maintained as heterozygotes using a balancer chromosome…"

© 2023. Published by The Company of Biologists under the terms of the Creative Commons Attribution License (https://creativecommons.org/licenses/by/4.0/).

It would be much clearer to say that the syb2172 allele is maintained over a sin-3(+) balancer chromosome, and syb2172 homozygotes were derived from than strain.

3) Correct typographical and spelling errors throughout the manuscript. For example: page 6, line 4 refers to “Y67D2.7 5” as an ORF? This ORF does not come up in a Wormbase search….

4) Page 11, lines 1-2: Include some description of the hypergeometric tests to the Methods section.

5) Page 12 and Fig. 4 legend: For clarity, please indicate that immunolabeling against active RNA pol II was performed. Also, immunolabeling is technically not the same as “staining” – such as with DAPI, for example - and newer members of the field would benefit from the authors making a clear distinction.

Reviewer 2

Advance summary and potential significance to field

The data in the article supports the summary in the abstract. The authors addressed my initial review of the article.

Comments for the author

I have one request from the authors, and it is to change "gender" to "sex" in fig 3F, as C. elegans has two sexes, not genders.

Second revision

Author response to reviewers' comments

Reviewer 1 Advance Summary and Potential Significance to Field:
As indicated in my review of the initial submission, this manuscript investigates the importance of SIN-3 histone deacetylase complexes in C. elegans development focusing particularly on the germ line. The study addresses a conserved process regulating gene expression in development and will be of general interest to readers. The authors provide evidence for two distinct SIN-3 complexes in C. elegans and identify sets of mRNAs whose expression levels are altered in the absence of SIN-3 activity.

Reviewer 1 Comments for the Author:
Resubmitted ms from Robert, Caron et al., "SIN3 acts in distinct complexes to regulate the germline transcriptional program in C. elegans"

In this resubmitted manuscript, the authors have addressed many of the comments from the three reviewers regarding the initial submission. The manuscript would benefit from the authors addressing a few more points.

Comments on the current version

1) Fig. S3: The authors state on page 7 that mutations in athp-1, hda-3, and suds-3 have "no obvious defects" to account for reduced brood size, and they provide images in Fig. S3 as evidence. However, these germlines in fact do not look normal. The athp-1 germline has tightly stacked oocytes and a few apparently unfertilized oocytes in the uterus; either this animal is depleted of sperm (early) or did not make sperm. The hda-3 germline has a set of small, atypical cells located at about the spermatheca; it is unclear if these are abnormal spermatocytes and/or abnormal oocytes. This phenotype resembles the "gogo" phenotype shown in Fig. S1B. The suds-3 germline appears very small, although the distal end may be obscured by the intestine.

© 2023. Published by The Company of Biologists under the terms of the Creative Commons Attribution License (https://creativecommons.org/licenses/by/4.0/).

We agree that hda-3 and athp-1 germlines are not fully wildtype, but we did not further analyze these more subtle phenotypes in the present study. We have instead nuanced our statement:
These results correlate with minor morphological defects observed in hda-3, athp-1 mutant germlines.
As the reviewer points out, the suds-3 germline appears smaller because the distal end is obscured by the intestine. ***We looked at more suds-3 germline images and could not find any obvious and significant morphological defects compared to wild-type.***

2) Page 4, top several lines: The authors state that tm1276 is a deletion of exon 3. It is important that the authors also tell us if this an in-frame deletion. If so, then this might help to explain why tm1276 has a greater impact on the transcriptome than does syb2172 (discussed on page 8). For example, the deleted protein may have residual and/or unregulated activity that alters gene expression.
The reviewer is correct, but for the time being we cannot distinguish between the different possibilities. An initial RNA-seq experiment performed on embryos suggested that** tm1276 **could potentially produce an in-frame deletion encoding a protein with partial function. However, reads from our germline RNA-seq suggest that the transcripts produced may be different in the germline. Since we did not carry out qRT-PCR, we cannot make predictions on the protein produced in this mutant. Given this ambiguity, we prefer to now state:

"The difference in the total number of misregulated genes may be attributed to differences in sample preparation and processing, or the specific allele used", rather than
"The difference in the total number of misregulated genes may be attributed to the specific allele used, or differences in sample preparation and processing."

Indeed as we discussed in detail in our rebuttal letter, experimental conditions were different in many ways between the two RNA-seq experiments.

In addition, the description of syb2172 strain maintenance is confusing. The authors state that "...syb2172 homozygous animals were maintained as heterozygotes using a balancer chromosome..." It would be much clearer to say that the syb2172 allele is maintained over a sin-3(+) balancer chromosome, and syb2172 homozygotes were derived from than strain.

We replaced the sentence "syb2172 homozygous animals were maintained as heterozygotes using a balancer chromosome" with "The syb2172 allele is maintained over a sin-3(+) balancer chromosome (see Material and Methods)."
3) Correct typographical and spelling errors throughout the manuscript. For example: page 6, line 4 refers to "Y67D2.7 5" as an ORF? This ORF does not come up in a Wormbase search....
"Y67D2.7 5" was replaced by "Y67D2.7"

4) Page 11, lines 1-2: Include some description of the hypergeometric tests to the Methods section.
All numbers used for hypergeometric tests performed in Figure 3D and Figure 4A are presented in the legend. For Figure 4B, numbers are given in Table S3.

5) Page 12 and Fig. 4 legend: For clarity, please indicate that immunolabeling against active RNA pol II was performed. Also, immunolabeling is technically not the same as "staining" – such as with DAPI, for example - and newer members of the field would benefit from the authors making a clear distinction.
Page 12: The sentence "Polymerase II (pol II) staining instead revealed a slight but reproducible increase on the X..." was replaced by "Immunolabelling of the active form of Polymerase II (pol II) instead revealed a slight but reproducible increase on the X....".
"staining" was replaced by "labeling" whenever necessary throughout the manuscript, including Fig. 4 legend.

Reviewer 2 Advance Summary and Potential Significance to Field:
The data in the article supports the summary in the abstract. The authors addressed my initial review of the article.

Reviewer 2 Comments for the Author:

© 2023. Published by The Company of Biologists under the terms of the Creative Commons Attribution License (https://creativecommons.org/licenses/by/4.0/).

I have one request from the authors, and it is to change "gender" to "sex" in fig 3F, as C. elegans has two sexes, not genders.
This was done.

Third decision letter

MS ID#: DEVELOP/2023/201755

MS TITLE: SIN3 acts in distinct complexes to regulate the germline transcriptional program in C. elegans

AUTHORS: Valerie J Robert, Matthieu Caron, Loic Gely, Annie Adrait, Victoria Pakulska, Yohann Coute, Manon Chevalier, Christian G Riedel, Cecile Bedet, and Francesca Palladino

Thank you for making all the changes to better enhance the clarity of the paper. In the revised manuscript there are two instances of the usage of the word "gender". I ask that you change this to "sex", which was originally pointed out by reviewer 2 in the last submission.

Third revision

Author response to reviewers' comments

Responses to reviewers:

Reviewer 1 Advance Summary and Potential Significance to Field:

As indicated in my review of the initial submission, this manuscript investigates the importance of SIN-3 histone deacetylase complexes in C. elegans development focusing particularly on the germ line. The study addresses a conserved process regulating gene expression in development and will be of general interest to readers. The authors provide evidence for two distinct SIN-3 complexes in C. elegans and identify sets of mRNAs whose expression levels are altered in the absence of SIN-3 activity.

Reviewer 1 Comments for the Author:
Resubmitted ms from Robert, Caron et al., "SIN3 acts in distinct complexes to regulate the germline transcriptional program in C. elegans"

In this resubmitted manuscript, the authors have addressed many of the comments from the three reviewers regarding the initial submission. The manuscript would benefit from the authors addressing a few more points.

Comments on the current version

1) Fig. S3: The authors state on page 7 that mutations in athp-1, hda-3, and suds-3 have "no obvious defects" to account for reduced brood size, and they provide images in Fig. S3 as evidence. However, these germlines in fact do not look normal. The athp-1 germline has tightly stacked oocytes and a few apparently unfertilized oocytes in the uterus; either this animal is depleted of sperm (early) or did not make sperm. The hda-3 germline has a set of small, atypical cells located at about the spermatheca; it is unclear if these are abnormal spermatocytes and/or abnormal oocytes. This phenotype resembles the "gogo" phenotype shown in Fig. S1B. The suds-3 germline appears very small, although the distal end may be obscured by the intestine.
We agree that hda-3 and athp-1 germlines are not fully wildtype, but we did not further analyze these more subtle phenotypes in the present study. We have instead nuanced our statement:

© 2023. Published by The Company of Biologists under the terms of the Creative Commons Attribution License (https://creativecommons.org/licenses/by/4.0/).

These results correlate with minor morphological defects observed in hda-3, athp-1 mutant germlines.
As the reviewer points out, the suds-3 germline appears smaller because the distal end is obscured by the intestine. We looked at more suds-3 germline images and could not find any obvious and significant morphological defects compared to wild-type.

2) Page 4, top several lines: The authors state that tm1276 is a deletion of exon 3. It is important that the authors also tell us if this an in-frame deletion. If so, then this might help to explain why tm1276 has a greater impact on the transcriptome than does syb2172 (discussed on page 8). For example, the deleted protein may have residual and/or unregulated activity that alters gene expression.
The reviewer is correct, but for the time being we cannot distinguish between the different possibilities. An initial RNA-seq experiment performed on embryos suggested that tm1276 could potentially produce an in-frame deletion encoding a protein with partial function. However, reads from our germline RNA-seq suggest that the transcripts produced may be different in the germline. Since we did not carry out qRT-PCR, we cannot make predictions on the protein produced in this mutant. Given this ambiguity, we prefer to now state:

"The difference in the total number of misregulated genes may be attributed to differences in sample preparation and processing, or the specific allele used", rather than
"The difference in the total number of misregulated genes may be attributed to the specific allele used, or differences in sample preparation and processing."

Indeed as we discussed in detail in our rebuttal letter, experimental conditions were different in many ways between the two RNA-seq experiments.

In addition, the description of syb2172 strain maintenance is confusing. The authors state that "...syb2172 homozygous animals were maintained as heterozygotes using a balancer chromosome..." It would be much clearer to say that the syb2172 allele is maintained over a sin-3(+) balancer chromosome, and syb2172 homozygotes were derived from than strain.

We replaced the sentence "syb2172 homozygous animals were maintained as heterozygotes using a balancer chromosome" with "The syb2172 allele is maintained over a sin-3(+) balancer chromosome (see Material and Methods)."
3) Correct typographical and spelling errors throughout the manuscript. For example: page 6, line 4 refers to "Y67D2.7 5" as an ORF? This ORF does not come up in a Wormbase search....
"Y67D2.7 5" was replaced by "Y67D2.7"

4) Page 11, lines 1-2: Include some description of the hypergeometric tests to the Methods section.
All numbers used for hypergeometric tests performed in Figure 3D and Figure 4A are presented in the legend. For Figure 4B, numbers are given in Table S3.

5) Page 12 and Fig. 4 legend: For clarity, please indicate that immunolabeling against active RNA pol II was performed. Also, immunolabeling is technically not the same as "staining" – such as with DAPI, for example - and newer members of the field would benefit from the authors making a clear distinction.
Page 12: The sentence "Polymerase II (pol II) staining instead revealed a slight but reproducible increase on the X..." was replaced by "Immunolabelling of the active form of Polymerase II (pol II) instead revealed a slight but reproducible increase on the X....".
"staining" was replaced by "labeling" whenever necessary throughout the manuscript, including Fig. 4 legend.

Reviewer 2 Advance Summary and Potential Significance to Field:
The data in the article supports the summary in the abstract. The authors addressed my initial review of the article.

Reviewer 2 Comments for the Author:

© 2023. Published by The Company of Biologists under the terms of the Creative Commons Attribution License (https://creativecommons.org/licenses/by/4.0/).

I have one request from the authors, and it is to change "gender" to "sex" in fig 3F, as C. elegans has two sexes, not genders.
This was done.

Fourth decision letter

MS ID#: DEVELOP/2023/201755

MS TITLE: SIN3 acts in distinct complexes to regulate the germline transcriptional program in C. elegans

AUTHORS: Valerie J Robert, Matthieu Caron, Loic Gely, Annie Adrait, Victoria Pakulska, Yohann Coute, Manon Chevalier, Christian G Riedel, Cecile Bedet, and Francesca Palladino
ARTICLE TYPE: Research Article

I am happy to tell you that your manuscript has been accepted for publication in Development, pending our standard ethics checks.

© 2023. Published by The Company of Biologists under the terms of the Creative Commons Attribution License (https://creativecommons.org/licenses/by/4.0/).
